# Parenting and stimulation for child development at home: perspectives from Nurturing Care*

**DOI:** 10.1590/1518-8345.7554.4611

**Published:** 2025-08-18

**Authors:** Jeniffer Stephanie Marques Hilário, Letícia Pancieri, Regina Aparecida Garcia de Lima, Elsa Maria Oliveira Pinheiro de Melo, Débora Falleiros de Mello

**Affiliations:** 1 Universidade de São Paulo, Escola de Enfermagem de Ribeirão Preto, PAHO/WHO Collaborating Centre for Nursing Research Development, Ribeirão Preto, SP, Brazil.; 2 Scholarship holder at the Conselho Nacional de Desenvolvimento Científico e Tecnológico (CNPq), Brazil.; 3 Universidade de Aveiro, Faculdade de Ciências da Saúde, Aveiro, Portugal.

**Keywords:** Child, Child Development, Child Care, Parenting, Nursing, Primary Health Care

## Abstract

to analyze maternal parenting practices aimed at the development of children under one year of age in the home context, from the perspective of the Nurturing Care framework.

a qualitative study, grounded in philosophical hermeneutics and the conceptual approach of Nurturing Care. In-depth, semi-structured interviews were conducted with 27 mothers, followed by inductive thematic analysis.

maternal parenting practices involved interaction and engagement in play activities with the child, socialization with other children, early exposure to screens, relative paternal participation, and occasional book reading. Uncertainties emerged regarding setting limits, early childhood education enrollment, and perceptions of child development. The findings indicated a partial interrelation with Nurturing Care domains related to responsive caregiving, opportunities for early learning, and child security and safety.

maternal parenting practices, analyzed through the lens of the Nurturing Care framework, reflected the child’s care environment, highlighting both strengths and vulnerabilities in promoting child development in the home context. The Nurturing Care domains are useful for child care within Primary Health Care, reinforcing the relevance of parental responsiveness and the child’s daily routine for their full development. This suggests a potential strategy to promote nursing care in child health.

## Highlights

**(1)** Daily home routine reflects parenting practices for child development. **(2)** Maternal reports express strengths and weaknesses of the child care environment. **(3)** A nurturing and interactive environment provides opportunities of child learning. **(4)** Daily constraints expose children to developmental vulnerabilities. **(5)** The Nurturing Care framework offers analytical domains of child care at home.

## Introduction

During early childhood, which encompasses the first six years of life, there is intense brain development, marked by the formation of structures that are highly influenced by interactions between caregivers and the child, as well as by the environments in which the child is embedded^(1)^. Adequate care and a diverse range of experiences are therefore of vital importance^(2)^.

Providing favorable conditions for child development is more effective and cost-efficient than attempting to mitigate the effects of adversity at a later stage^(3)^. In terms of public policy, efforts have been directed toward promoting the full development of all children^(4)^. The 2030 Agenda, which comprises the 17 Sustainable Development Goals (SDGs), includes a focus on fostering child development, highlighting the necessity of universal access to health care services to ensure appropriate childhood care^(5)^.

In early childhood, achieving the Sustainable Development Goals (SDGs) is closely linked to ensuring Nurturing Care for children^(2)^. The Nurturing Care framework emphasizes holistic early childhood development and includes the following domains: good health, adequate nutrition, responsive caregiving, opportunities for early learning, and child security and safety^(2)^. However, studies have shown that this approach has been primarily focused on the health and nutrition components, with less attention to opportunities for early learning, responsive caregiving, and security and safety^(6-8)^, particularly concerning young children care in low- and middle-income countries. Scientific evidence highlights the importance of developmental surveillance and monitoring of early childhood milestones, identifying adverse conditions, and analyzing both short- and long-term consequences^(9-10)^.

The need to prioritize the development of children under one year of age is emphasized, given the likelihood of developmental issues arising in the early years of life^(11)^. Likewise, interventions aimed at improving outcomes for parental caregivers and supporting child development require further advancements^(12)^. Moreover, pediatric nursing care holds significant potential for assessing child health and development, as well as for implementing timely interventions. These are essential components of primary health care and are inherently linked to the clinical competencies in this field^(13-14)^.

Given the contemporary challenges, particularly in low- and middle-income countries, including Brazil, it is scientifically relevant to focus on parenting practices for children in their first year of life. This approach highlights child development in terms of responsive caregiving, opportunities for early learning, and security and safety based on maternal perceptions, which are central to the present investigation. In this regard, the study aims to understand parenting practices within the home care environment, explore the potential of the Nurturing Care framework, and examine its implications for pediatric nursing care in the field of Primary Health Care (PHC). Thus, the objective of this study is to analyze maternal parenting practices aimed at the development of children under one year of age in the home context, from the perspective of the Nurturing Care framework.

## Method

### Study design

This is a qualitative study based on the perspective of Gadamerian hermeneutics^(15)^, anchored in a way of conceiving and establishing relationships with others. This approach is based on a process in which dialogue serves as the *locus* of communication^(16)^, emphasizing the understanding of participants’ situations and experiences. The research focuses on maternal perceptions of the care of children under one year old in the home context, with an emphasis on child development, and is grounded in the conceptual framework of Nurturing Care^(2)^.

The study followed the guidelines of the Consolidated Criteria for Reporting Qualitative Research (COREQ)^(17)^.

### Location

The study was conducted in the municipality of Itaú de Minas, Minas Gerais, Brazil, within the coverage area of the Family Health Strategy (FHS) units. It is a small municipality with an estimated population of 16,286 inhabitants^(18)^, and has a total of five health units, all operating under the FHS. These five units cover the urban area, while one also serves the rural area of the municipality, which was not included in the study’s target population.

### Period

The research was conducted in the municipality from May 2 to July 31, 2023.

### Selection criteria

The inclusion criteria were: women aged 18 years or older, who had a low-risk pregnancy, were registered and receiving follow-up care at FHS units, lived in a household, and had children up to one year old. The exclusion criterion was: children with special needs. Information was obtained from health unit records. Participants who could not be located after three attempts to conduct a home visit (HV) were considered losses.

### Participants

The five FHS units covered a total of 114 women with children under one year of age. The inclusion and exclusion criteria were verified, along with an assessment of the distribution across the units, identifying the follow-up in child care for children under 12 months. In the end, 41 women met the criteria. Children under one year old, born between 05/02/2022 and 04/30/2023, were included. Regarding the distribution across health units, the final sample included 10 participants from the FHS-1 area, five from FHS-2, five from FHS-3, four from FHS-4, and three from FHS-5.

### Instruments used for data collection

Data collection was conducted through semi-structured interviews, which included the characterization of the mothers (age, education level, presence of the child’s father or partner, family income, and maternal employment) and the children (age and sex), and the guiding question: “*What have the daily routines of stimulation and learning been like in caring for <child’s name>?*”.

### Data collection

The initial contact with the interviewees took place at health care units to explain the purpose of the study, the data collection procedures, and to obtain their consent for a home visit. Initially, two pilot interviews were conducted to assess the adequacy of the script and identify possible adjustments; however, these interviews were later excluded, as additional questions were included.

The interviews were conducted in person and individually through HV, recorded in audio after obtaining written informed consent. A guiding question was used to initiate the interview, followed by a sequence of additional questions, forming a 24-hour daily activity map of interactions with the child, particularly during the morning, afternoon, and evening periods. This approach aimed to establish a hermeneutic perspective in the interaction with the interviewees.

It was assumed that illustrating the child’s routine based on maternal perceptions would allow for a comprehensive analysis of child care, developmental stimuli, and monitoring in the home environment. These aspects guided the formulation of additional questions linked to the Nurturing Care domains. Each interview lasted an average of 50 minutes and was conducted by the first author. Field notes were not taken.

The decision to discontinue the interviews was based on the composition of participants’ narratives, considering the uniqueness, depth, and heterogeneity of qualitative data^(19)^, particularly regarding child development and the conceptual approach adopted, relevant to the study’s objective.

### Data processing and analysis

The data analysis was based on thematic analysis, following an inductive approach. The analysis process, focused on the experiences shared by the participants during the interviews, was supported by a hermeneutic attitude, as a movement of understanding knowledge and discoveries about the other and with the other, and guided by the principles of child care within the Nurturing Care framework.

The thematic analysis followed the steps of initial familiarization with the data, repeated readings of the empirical material, generation of codes, theme designation, and descriptive and qualitative interpretation^(20)^.

The full transcription of the interviews was carried out by the first author, with the organization of individual digital files in Microsoft Word^®^. These files were then imported into the Atlas.ti^®^ software to manage the data and code the collected information.

The coding performed by Atlas.ti^®^ generated elements that were discussed by the authors, who validated the thematic units and themes derived from the participants’ narratives, ensuring the reliability of the study. The participants were identified with the letter M (M1, M2, M3...) and the children with the letter C (C1, C2, C3...), followed by their age in months.

## Ethical aspects

The original study project received a favorable opinion from the Research Ethics Committee, with Certificate of Presentation for Ethical Consideration number 6,056,891.

## Results

The results present the maternal parental reports of 27 women, whose characteristics are described in Table 1.

**Table 1-:** Characteristics of the participating mothers and their children. Itaú de Minas, MG, Brazil, 2023

Sociodemographic characteristics		n	%
Maternal age	18 to 25 years	11	42
	26 to 35 years	11	42
	36 years or older	5	16
Children’s age	0-3 months	3	11
	4-6 months	9	33
	7-9 months	9	33
	10-11 months	6	23
Geographic area of residence	Urban	27	100
	Rural	0	0
Education level	Completed high school	14	52
	Incomplete high school	8	30
	Incomplete elementary school	4	15
	Completed university education	1	3
Relationship status	With partner	24	89
	Without partner	3	11
Child’s father lives in the same house	Yes	22	81
	No	5	19
Works outside the home	Yes	6	22
	No	21	78
Monthly income	< 1 MW*	4	15
	1-2 MW*	20	74
	≥ 3 MW*	3	11

*MW = Minimum wage in Brazil in 2023, amounting to R$1,320.00

A significant proportion of the mothers were young, mostly with a high school diploma and dedicated to domestic work. The majority reported a household income below three minimum wages (MW), based on the 2023 value (R$ 1,320.00)^(21)^. In this context, half of the participants stated that they did not face economic difficulties, while others reported experiencing some financial needs. All fathers or partners had jobs outside the home.

The children’s ages ranged from two to 11 months. Regarding the father’s residence in the same household, most mothers lived with the children’s biological fathers. 

**Figure 1-:**
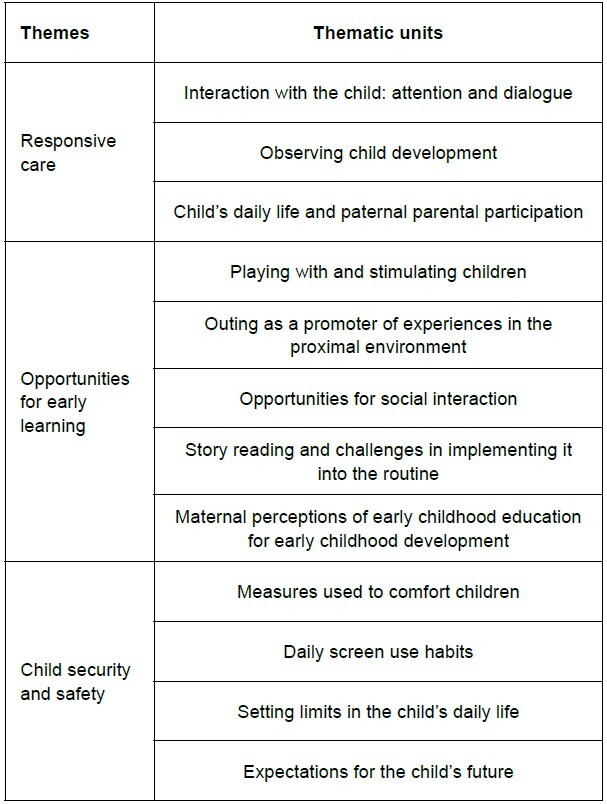
Themes and thematic units of the results. Ribeirão Preto, SP, Brazil, 2024

The qualitative results were organized into themes aligned with the studied domains of Nurturing Care. Figure 1 presents the themes and their corresponding thematic units.

The thematic unit *Interaction with the child: attention and dialogue* explores accounts of how mothers interact and communicate with their children*. She pays attention to everything I say. So, I think it is really good; she will learn by watching, feeling, and experiencing* (M27 C27, 7 months). *Talking is part of their development, how else will they understand what we are saying? In practice, we talk to them, show them things, and explain what they are* (M25 C25, 5 months). On the other hand, some mothers do not recognize the impact of maternal speech on child development, arguing that the child neither understands nor focuses on the conversation. *Talking depends on age, right? It is good for learning, but at her age, I do not think it is very effective because she does not even pay attention* (M6 C6, 4 months).

The thematic unit *Playing with and stimulating children* encompasses daily routines, attention, and play as essential elements for child development. *Routine, first and foremost, is necessary; otherwise, we cannot manage. Besides routine, affection and attention, I think those should never be lacking* (M4 C4, 8 months). *Playing with him more, showing him more things. Showing him the world and teaching him how to live here, how to adapt to life’s storms* (M13 C13, 7 months). Participants describe different types of play activities with their children and how they perceive play as a means of parent-child interaction. *She plays hide and seek a lot with me and my husband. She even hides by herself now. We talk to her a lot, tickle her, and play by using her stuffed animals to ‘talk’ to her* (M3 C3, 10 months). The accounts also provide examples of playful moments during child care routines, highlighting concerns about potential household accidents. *During bath time, he plays a lot, splashing his hands in the water, playing with his toys, his little duck, and even throwing water at me, it is a party!* (M17 C17, 9 months). *He constantly tries to grab the shampoo or conditioner bottle. I also give him a rubber duck. If I am not careful, he might even drink the dirty bathwater, I have to keep a close eye on him* (M24 C24, 8 months).

The thematic unit *Observing child development* highlights maternal apprehension of development through the signs children exhibit, their movements, and their interaction with the environment. *She already knows how to open and close her hands, if I call her, she responds and comes to me, she already knows where her feet, hands, and nose are* (M1 C1, 10 months)*. She looks at us and shows that she sees the kitten. Before, she was not aware of anything, but when I started talking to her and showing her the kitten, for example, I could see that she began to recognize it* (M6 C6, 4 months). Other situations were mentioned as concerning when certain developmental milestones were not reached according to age. *He does not crawl yet, he spends very little time in the walker, compared to my other children, he is more sluggish, he says ‘quê’ but has not learned to pronounce anything yet, although he screams a lot* (M10 C10, 11 months).

The thematic unit *Child’s daily life and paternal parental participation* addresses the role of the father as a caregiver, focusing on his interaction and involvement in child care. Positive paternal involvement is reported through play and the performance of some caregiving tasks. *The father is crazy about her; he plays a lot, stays with her while I take a shower, changes her diaper, and feeds her. Most days, he stays with her at night* (M1 C1, 10 months). Paternal care is compared to maternal care: *His father is a completely different version of me. He is attentive and calm with the boys* (M11 C11, 3 months). The time fathers spend with their children is described as limited, often framed as assisting the mother*. He is very helpful, and when he gets home at four in the afternoon, he takes care of the kids, bathes the older one, plays with the baby while I make dinner, and occasionally changes diapers* (M4 C4, 8 months). *He participates when he is here. He takes care of the baby while I cook lunch and dinner* (M7 C7, 8 months). From the maternal point of view, little paternal involvement is associated with limited time with the child, and is perceived by mothers as a dilemma. *He does things his way. He comes home exhausted, plays for a little while, and then quickly hands the baby back to me* (M5 C5, 11 months). *He stays with her for a bit after work. But changing diapers, bathing, and putting her to sleep, that is all on me* (M21 C21, 8 months). *He spends the whole day at work. Then he comes home tired and does not have much contact with her* (M13 C13, 7 months).

The maternal interviews revealed that 14 fathers or partners participated in child care, while 13 did not. Among the 27 women, 25 received support from non-parental caregivers, including 20 from the child’s grandmother, three from siblings over the age of 20, one from an aunt, and one from a babysitter. When the biological father did not live with the child, interaction was reported through visits and financial contributions. *He only participates financially. He calls me and sends messages asking if she is okay. He only sees her through video* (M12 C12, 9 months)

The thematic unit *Outing as a promoter of experiences in the proximal environment* addresses the opportunities provided to children outside the home, using outings as a form of distraction. *He likes going out, taking walks. We go to the little square, or when I pick up one of his siblings from school, I take him along* (M10 C10, 11 months). *I take him on many walks through the streets, put him in the stroller and walk with him, I have already taken him to the playground and to eat cotton candy at the little square, he loves it* (M23 C23, 6 months). Some reports mention difficulties with this type of activity, leading parents to avoid frequent outings. *He does not stop for a second, and it is very difficult to keep running after him, it is exhausting. So, we prefer to just let him play freely here at home* (M15 C15, 11 months). Concerns about potential exposure to illnesses were also highlighted. *I do not like taking her out too much, I am afraid of diseases* (M20 C20, 6 months).

The thematic unit *Opportunities for social interaction* highlights interactions with siblings and other children. *Her brother plays with her a lot, teasing, tickling, and playing peek-a-boo* (M21 C21, 8 months). Interaction with other children was also reported through the extended family. * I take her to my grandmother’s, there are lots of kids, our cousins. I go there at least once a week, she just watches and laughs at everything* (M6 C6, 4 months). *Only when I take him out for a walk, to the grandmothers’ house, then he sees the younger cousins* (M19 C19, 5 months). Relationships in the neighborhood and other environments were also mentioned, highlighting connection with interaction and stimulation. *There is the neighbor’s daughter, I always go there to talk to her and the two little ones ‘talk’ too* (M9 C9, 5 months). *If I go outside with her, she wants to see everyone, waves, laughs, does a ‘thumbs up’ with her hand. She plays with the puppy, touches it, pulls its paw* (M12 C12, 9 months).

The thematic unit *Story reading and challenges in implementing it into the routine* explores how reading is conducted for children and mothers’ perceptions of this activity. Regarding reading moments, mothers reported engaging with their children by reading, talking, and showing pictures. *He has his little books, I lay them on the mattress and show him the pictures, talking about animals, fruits, and pointing things out to him* (M10 C10, 11 months). Some also associate reading with religious teachings. *I have told him stories, but mostly from the Bible. I talk a lot about God and Joseph. I talk to him a lot* (M5 C5, 11 months).

Having children’s books at home does not necessarily mean they are used, as mothers pointed out challenges due to a lack of interest from the child. *Just yesterday, I tried. But she does not pay attention. I think she does not understand yet* (M3 C3, 10 months). *There are several books around the house. Reading to her is tricky because she does not sit still to listen* (M12 C12, 9 months). Other difficulties emerged, such as the mothers’ lack of reading habits and the perception that children do not understand due to their age. *It is not a habit of mine, so I do not take the initiative, and she does not pay attention for long* (M1 C1, 10 months). *We do not have any little books, but I will see if I can buy some to show her the pictures. But I think she will only start understanding after a year old. Right now, it seems like she does not get it at all* (M7 C7, 8 months).

The thematic unit *Maternal perceptions of early childhood education for early childhood development* explores aspects of enrolling children in daycare. Among the participants, 17 intended to enroll their child at the age of two, following the criteria set by the municipality where the study was conducted. Favorable perceptions highlight the benefits of early childhood education. *She will have contact with other children and people, which will contribute to her development, studies say that* (M6 C6, 4 months). *It will help, right? Because there will be other children for her to play and interact with. She will learn from an early age to share space and to speak in order to communicate what she wants* (M27 C27, 7 months). *There, they give more attention than we do at home. The caregivers are dedicated to the children, they sing, play, have nap time, everything is well organized* (M10 C10, 11 months). *They teach a lot there, right? She meets many children, has set times for eating, sleeping, everything, and this helps both me and her to follow a fixed routine* (M16 C16, 3 months).

Some mothers reported choosing not to enroll their children in daycare, justifying their decisions. *I do not intend to because I do not work, right? So, she will not go to daycare, not even when she is older, only when she has to go to school* (M3 C3, 10 months). *I do not intend to. I am afraid of diseases, and I also see so many reports of daycare centers mistreating children, I am afraid they might mistreat him* (M14 C14, 11 months).

The thematic unit *Measures used to comfort children* describes the strategies employed to stop crying and meet children’s needs. *To comfort her, I hold her, show her toys, and take her to the garden here at home. Sometimes, I put her in the stroller and take a walk around the block to try to distract her* (M7 C7, 8 months). *To comfort, I just hold her and breastfeed her* (M16 C16, 3 months). There was also a report of using medication to calm the child, highlighting potential uncertainties regarding assumed pain and the indiscriminate use of medication at home. *I give her dipyrone, hold her a lot, take her for a walk in the street, and distract her with the television* (M12 C12, 9 months).

The thematic unit *Daily screen use habits* relates to children’s routines for watching programs, with a particular focus on television. *Around 12:30 p.m., she watches TV with me. I feed her, and she falls asleep. In the afternoon, she wakes up, I bathe her, change her clothes, and leave the TV on to entertain her* (M6 C6, 4 months). *She watches TV practically all morning and a little in the afternoon. If I have something to do, there is no other way, I let her watch something while I keep an eye on her from a distance* (M10 C10, 11 months). There were mentions of perceived benefits of screen use for children. *Screens nowadays help us a lot, right?* (M1 C1, 10 months). *TV ends up helping a bit, it grabs the child’s attention and teaches a lot of things* (M26 C26, 4 months). Mobile phones were also mentioned as a way to comfort children, alternating with play. *To comfort her, we try playing with her, tickling her, or putting a cartoon on the phone* (M6 C6, 4 months).

The thematic unit *Setting limits in the child’s daily life* explores the forms and use of restrictions in children’s activities. The word “no”, repeated emphatically, is commonly mentioned as a way to establish boundaries, so that children understand and learn what is expected of them. *I usually say the dreaded “no”, the first time, calmly. But she is in the phase of insisting, so she goes back and tries again. Then, I change my tone of voice, speak firmly, and she gives up, pouts, but does not cry, and we distract her with something else* (M1 C1, 10 months). *I say “no” to her a lot, take her hand away from the outlet, and say no, making a serious face, then she pouts, trying to convince me to let her do it. So, I move her away and distract her* (M18 C18, 9 months). Changes in tone of voice and facial expressions are also mentioned as strategies to make children aware that they are doing something wrong. *To set limits, I speak firmly, make a stern or disappointed face. I think she is just starting to understand now (M6 C6, 4 months). I make a stern face, deepen my voice, and speak firmly, telling her she cannot do that, that it could hurt, that it is not for babies* (M9 C9, 5 months).

Another approach mentioned is using time-outs as a form of punishment in cases of disobedience. *I even put him in time-out the other day. He kept trying to put his toys in the outlets. I told him he could not, but he would not listen. So, I picked him up, put him in the crib, and told him he was in time-out* (M15 C15, 11 months). Setting limits is described as challenging, often justified by the child’s young age and perceived inability to understand. *He does not usually understand what I say, so I do not enforce many limits* (M13 C13, 7 months)*. She still does not understand much, so I just move her away or take things out of her hands when it is something she cannot have* (M26 C26, 4 months)*.*

The thematic unit *Expectations for the child’s future* reflects some maternal aspirations for their children’s lives, highlighting concerns about current social circumstances and the desire for divine protection. *We do not know what tomorrow holds. I get really worried when I see the state of the world today. How will this child’s life be? I hope God watches over her, that everything turns out well. That she becomes a wonderful person, right? Studious. But only God knows* (M2 C2, 6 months). Education is emphasized as a key factor in shaping positive future prospects. *I want her to study a lot, right? Study hard. I want her to study a lot. I think she has everything it takes to be very studious and intelligent* (M9 C9, 5 months). *I want her to study a lot and leave here, build her own life, have her own things* (M22 C22, 2 months). Certain socio-emotional traits, combined with the importance of education, are highlighted as aspirations for the child’s future. *I want him to be a good person, someone who knows how to get along with everyone despite differences, who can also achieve something in life, who is very studious* (M15 C15, 11 months). *I hope he is determined, a dreamer, but that he fights for what he wants, that he is intelligent and studies hard to have a good job, have his own things, and maybe even a family if he wants to get married* (M8 C8, 6 months).

**Figure 2-:**
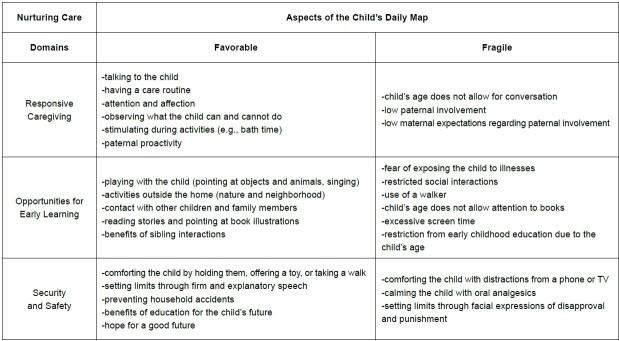
Nurturing Care domains related to aspects of the child’s daily map. Ribeirão Preto, SP, Brazil, 2024

The set of results was analyzed and interrelated with the domains of Nurturing Care, highlighting responsive caregiving, opportunities for early learning, and security and safety (Figure 2).

The results express components of the child’s routine based on maternal perceptions, including elements that both qualify the care environment and present weaknesses and uncertainties for child development in the home context and its surroundings.

## Discussion

The maternal parenting practices identified encompassed the child’s care environment routine, including aspects such as interacting and playing with the child, recognizing child development, identifying paternal involvement, socializing with other children, reading books, screen use, early childhood education, comforting measures, setting boundaries, and future expectations. This set of elements has implications for child development and is interrelated with the Nurturing Care domains of responsive caregiving, opportunities for early learning, and the child’s security and safety in their first year of life.

Maternal reports highlight various forms of parental interaction, including attentiveness to the child and observing whether they are developing according to their age. During early childhood, parental interactions serve as a fundamental basis for development, particularly in memory, flexibility, and creativity^(2,22)^. Learning at home, primarily shaped by parental interactions, has a powerful and positive influence on early childhood development outcomes compared to other life stages, due to the rapid pace of early brain development^(23)^. Positive environments can impact children’s development while also benefiting parental mental health^(24)^.

Maternal parenting practices highlight that interactions with other children occur within the family, neighborhood, and friends, providing stimulation opportunities through shared play, eliciting smiles, observing, pointing and showing objects, fostering interaction and learning. Parental engagement in play activities benefits child development^(25)^. A broad range of stimulation is essential, encompassing sensory experiences, the child’s potential, and adult-child interaction^(26)^. Playing with household items, such as empty plastic and cardboard containers, aids learning by exposing children to different textures, shapes, and colors, encouraging active engagement^(27)^. “Peek-a-boo” interactions help develop motor, socioemotional, cognitive, and language skills while also reducing behavioral issues in early childhood^(6,25)^. Additionally, parental knowledge of child development is crucial for recognizing milestones and specific needs essential for a child’s progress^(28)^.

The reading of children’s storybooks was mentioned in maternal reports as a positive activity, but doubts arose about the appropriate age and the child’s lack of interest. In this regard, other studies have identified that owning children’s books is more strongly associated with parental interactions and language development compared to routines of playing and singing^(29-30)^. On the other hand, one study found that parental caregivers rarely engaged in reading stories with children^(31)^.

In the present study, taking children for walks was seen as a moment to unwind and provide them with new experiences of exploring different environments. Some reports expressed concerns about disease exposure, the child’s behavior, and issues related to public breastfeeding. Outdoor play has been linked to opportunities for children to develop independence, self-determination, and physical skills^(32)^, as well as social, emotional, and cognitive benefits, including positively challenging elements^(33)^.

Screen use was associated with mothers’ ability to carry out household tasks, with television or mobile phones used to entertain the child. Notably, children under one year old were reported to use mobile devices, highlighting the need for parental guidance on potential developmental risks and promoting appropriate screen use for digital well-being. Studies have shown that excessive exposure to digital media is linked to developmental and behavioral issues in young children^(34-35)^. Another study emphasized that children with less screen time engaged more with their caregivers^(29)^.

Holding and providing distractions in the child’s care environment were mentioned as common comforting practices, stimulating other senses and calming the child. Other methods to shift focus from crying included play, walks, breastfeeding/feeding, and offering distractions to reduce discomfort. A similar aspect was highlighted in a study on parental caregivers who used distraction techniques to soothe and prevent prolonged crying episodes in infants^(36)^.

In the reported maternal practices, setting limits was seen as a way to teach conduct, sometimes as a form of reprimanding children’s actions. Considerations regarding the child’s age highlighted references to the developmental process. Another study^(37)^ indicated that parental caregivers felt unprepared to handle challenging behaviors, such as tantrums, often resorting to yelling and swearing. Adverse childhood experiences are linked to negative parental attitudes, leading to greater developmental vulnerabilities when parenting is less affectionate and more severe^(38)^. This underscores the importance of fostering protective childhood experiences, reducing hostile, aggressive, and coercive behaviors, which are key predictors of family violence, and promoting positive parenting changes^(39)^. Additionally, research emphasizes distinguishing between parenting practices (inductive, coercive), parenting styles (authoritarian, indulgent, negligent), and parental educational social skills (expressing affection, attention, opinions, and rights while minimizing punishment), broadening the scope for professional interventions in child care^(40)^.

The results also highlight the need for preventing household accidents during the first year of life, emphasizing the importance of integrating safety measures with interaction, dialogue, and boundary-setting. Young children are more vulnerable to domestic injuries due to their ongoing development of physical and social skills, requiring a safe and stimulating learning environment with preventive measures at home^(41)^. Moreover, parental factors in unintentional injuries should be considered, including parental mental health challenges, low paternal involvement in child care, single-parent households, disorganized home environments, among others^(42)^.

The inclusion of children in early childhood education raised maternal perspectives on its advantages and disadvantages, particularly regarding learning opportunities and social interaction for proper development. Concerns, uncertainties, and fears were also expressed about the quality of the environment and the role of educators. Mothers emphasized education and study as pathways to success, alongside happiness and positive life choices. Religious values were highlighted as a way to believe that children would be protected and guided toward a promising future.

Most participants reported a household income below three minimum wages, and their expectations for their children’s future reflected concerns about their social circumstances. A study indicates that families relying heavily on non-parental caregivers, such as grandparents or others, express worries about the quality and accessibility of early education^(43)^. Preventive interventions within primary child health care, with universal access and strong engagement, have been shown to reduce disparities and help understand how poverty can create barriers to healthy relationships between mothers, fathers, and children^(44)^. Decision-making regarding alternative caregiving by different individuals and institutions can have both positive and negative impacts on early childhood development^(45)^.

In this study, the findings on maternal parenting practices provided qualitative insights into child development, which are interrelated with the domains of Nurturing Care, particularly responsive caregiving, opportunities for early learning, and child security and safety, with a focus on the first year of life. These domains must be strengthened within health care services to ensure high-quality, person-centered care that is equitable, resilient, and efficient^(46)^. Therefore, parental interventions in health care services aimed at preventing harm and promoting child development are crucial in the first three years of life^(6,47-48)^. Programs focused on mitigating early disadvantages play a key role in fostering responsive stimulation and appropriate learning opportunities^(49)^. Additionally, strategies within the primary care system should be re-evaluated to address structural barriers, organizational challenges, and financial constraints^(50)^.

This study presents implications for nursing care in child health within PHC, based on findings about parental practices at home, emphasizing the development of children under one year of age, a phase marked by significant changes when the developing brain is most sensitive to experiences and the environment.

Nursing care in child health often involves identifying, managing, and intervening in situations that encompass: (i) responsive parental care, to enhance conversations, attention, and affection with the child, assess care routines, observe developmental milestones, encourage paternal proactivity, and identify parental concerns about development; (ii) age-appropriate early learning, to stimulate through play, reading, songs, and outings, while promoting interactions with other children; and (iii) child security and safety, to balance comforting the child with setting limits, prevent household accidents, and understand the benefits of quality early childhood education. Thus, supporting responsive caregiving and age-appropriate learning, as well as intervening for child security and safety, represent a promising and qualified professional approach.

In this study, attention to daily routine situations helps identify vulnerabilities and address them based on the Nurturing Care domains. Therefore, timely, creative, and health-promoting interventions for child development must be intrinsic to nursing care in child health, fostering partnerships and shared parental responsibility to mitigate weaknesses and enhance an appropriate environment for comprehensive child care and development. The qualitative elements analyzed also highlight the need to strengthen continuity of care, which is essential in child health monitoring, home visits, prenatal care, and child care management - key areas of nursing care in PHC.

The study’s limitations include interviews conducted exclusively with mothers and a focus on the first year of children’s lives, based on the specific context of a small municipality with FHS units. Although the Nurturing Care domains related to good health and adequate nutrition were not addressed, the results highlighted the relevance of responsive caregiving, opportunities for early learning, and security and safety in the first year of life, emphasizing both strengths and vulnerabilities in child development within the home environment. Further research is needed on the perceptions of other parental and non-parental caregivers, different age groups, and early childhood developmental challenges.

## Conclusion

Maternal parenting practices were analyzed with an emphasis on the perception of the child’s daily routine focused on child development, highlighting strengths and weaknesses in their everyday life. Additionally, the study expanded the understanding of the caregiving and interaction environment, which provides learning opportunities and daily limitations, suggesting exposing children to vulnerabilities at vital circumstantial moments for their developmental needs from an early age.

The Nurturing Care domains related to responsive caregiving, opportunities for early learning, and security and safety are crucial for a child’s first year of life, encompassing interaction experiences, various moments and actions within the home care environment. Focusing on the first year allows for an understanding of the dynamic nature of child development, indicating new ways to perceive and guide parental care. These domains help identify both vulnerabilities and strengths in caregiving, given their contingent nature, as they involve dealing with unpredictability, uncertainty, and the varying circumstances linked to early experiences.

The Nurturing Care approach offers domains of analysis in the field of PHC, reaffirming the relevance of considering parental responsiveness in child care and understanding their daily routine in aspects that are substantial to full development and comprehensive care, suggesting a strategy that promotes nursing care in child health.
