# Supplementary material for: Parenting and stimulation for child development at home: perspectives from Nurturing Care
Source: Rev Lat Am Enfermagem. 2025 Aug 18;33:e4611. doi: 10.1590/1518-8345.7554.4611 (PMC12364457; doi:10.1590/1518-8345.7554.4611)
Supplement: Translation

# Translation: Parentalidade e estímulo ao desenvolvimento infantil em domicílio: perspectivas do *Nurturing Care* *

**DOI:** 10.1590/1518-8345.7554.4612

**Authors:** Jeniffer Stephanie Marques Hilário, Letícia Pancieri, Regina Aparecida Garcia de Lima, Elsa Maria Oliveira Pinheiro de Melo, Débora Falleiros de Mello

**Affiliations:** 1 Universidade de São Paulo, Escola de Enfermagem de Ribeirão Preto, Centro Colaborador da OPAS/OMS para o Desenvolvimento da Pesquisa em Enfermagem, Ribeirão Preto, SP, Brasil.; 2 Bolsista do Conselho Nacional de Desenvolvimento Científico e Tecnológico (CNPq), Brasil.; 3 Universidade de Aveiro, Faculdade de Ciências da Saúde, Aveiro, Portugal.

**Keywords:** Criança, Desenvolvimento Infantil, Cuidado Infantil, Parentalidade, Enfermagem, Atenção Primária à Saúde

## Abstract

analisar práticas parentais maternas voltadas ao desenvolvimento de crianças menores de um ano de idade no contexto domiciliar, na perspectiva do *Nurturing Care*.

estudo qualitativo, amparado na hermenêutica filosófica e abordagem conceitual do *Nurturing Care*. Foram realizadas entrevistas semiestruturadas em profundidade com 27 mães e análise temática indutiva.

as práticas parentais maternas envolveram interação e brincadeiras com a criança, convívio com outras crianças, introdução do uso de telas desde tenra idade, relativa participação paterna e leitura de livros de forma eventual. O estabelecimento de limites, a inserção na educação infantil e a percepção do desenvolvimento infantil destacaram-se por suas incertezas. O conjunto dos resultados indicou inter-relação parcial com domínios do *Nurturing Care* para cuidados responsivos, aprendizagem oportuna, segurança e proteção infantil.

as práticas parentais maternas, a partir da abordagem do *Nurturing Care*, refletiram o ambiente de cuidado da criança, sinalizando fortalezas e vulnerabilidades para promover o desenvolvimento infantil em contexto domiciliar. Os domínios do *Nurturing Care* são úteis para o cuidado infantil na Atenção Primária à Saúde, reafirmando a relevância da responsividade parental e da rotina diária da criança para o seu desenvolvimento pleno, sugerindo uma estratégia promotora do cuidado de enfermagem em saúde da criança.

## Destaques

**(1)** Rotina diária no domicílio retrata práticas parentais voltadas ao desenvolvimento infantil. **(2)** Relatos maternos expressam qualidades e fragilidades do ambiente de cuidado infantil. **(3)** Um ambiente de cuidado e interação proporciona oportunidades de aprendizado infantil. **(4)** Restrições no cotidiano expõem as crianças a vulnerabilidades desenvolvimentais. **(5)** A abordagem do *Nurturing Care* oferece domínios de análise do cuidado infantil em domicílio.

## Introdução

Na primeira infância, período envolvendo os seis primeiros anos de vida, ocorre intenso desenvolvimento cerebral, permeado pela formação de estruturas que recebem muitas influências das interações entre os cuidadores e a criança e em seus ambientes de inserção^(1)^, sendo de vital importância o cuidado adequado e um leque de experiências^(2)^.

O oferecimento de condições favoráveis ao desenvolvimento infantil é mais eficaz e menos dispendioso do que buscar posteriormente mitigar os efeitos das adversidades^(3)^. Em termos de políticas públicas, esforços vêm sendo direcionados para promover o desenvolvimento pleno de todas as crianças^(4)^. A Agenda 2030, composta pelos 17 Objetivos de Desenvolvimento Sustentável (ODS), inclui o direcionamento à promoção do desenvolvimento da criança, indicando a necessidade do acesso universal aos serviços de saúde para cuidados apropriados à infância^(5)^.

Na primeira infância, o alcance dos ODS está interligado à garantia do *Nurturing Care* para as crianças^(2)^. A abordagem do *Nurturing Care* enfatiza o desenvolvimento pleno na primeira infância, e é composta pelos seguintes domínios: boa saúde, nutrição adequada, cuidados responsivos, aprendizagem desde tenra idade, e segurança e proteção da criança^(2)^. No entanto, estudos indicaram que essa abordagem tem sido mais concentrada nos componentes de saúde e nutrição, com menos atenção à aprendizagem desde tenra idade, cuidados responsivos, e segurança e proteção^(6-8)^, particularmente quanto aos cuidados de crianças pequenas em países de baixa e média renda. Evidências científicas expressam a relevância da vigilância do desenvolvimento infantil e monitorização dos seus marcos, identificando suas condições adversas e analisando consequências a curto e longo prazo^(9-10)^.

A necessidade de priorizar o desenvolvimento em menores de um ano é destacada, visto que há probabilidade de uma criança ter problemas desenvolvimentais nos primeiros anos de vida^(11)^. Além disso, as intervenções para melhorar os resultados orientados aos cuidadores parentais e apoiar o desenvolvimento infantil precisam de avanços^(12)^. Ademais, o cuidado de enfermagem em saúde da criança tem muitas potencialidades para a avaliação da saúde e do desenvolvimento infantil e para a realização de intervenções oportunas, como elementos essenciais nos cuidados primários em saúde e inerentes às competências clínicas neste campo^(13-14)^.

Diante de desafios contemporâneos existentes particularmente nos países de baixa e média renda, incluindo o Brasil, há relevância científica para focar em práticas parentais voltadas às crianças no primeiro ano de vida, destacando o desenvolvimento infantil quanto aos cuidados responsivos, aprendizagem oportuna, e segurança e proteção a partir de percepções maternas, de interesse na presente investigação. Neste caminho, busca-se compreender as práticas no ambiente de cuidado domiciliar, as potencialidades do *Nurturing Care* e os desdobramentos para o cuidado de enfermagem em saúde da criança no campo da Atenção Primária à Saúde (APS). Assim, o objetivo do estudo é analisar práticas parentais maternas voltadas ao desenvolvimento de crianças menores de um ano de idade no contexto domiciliar, na perspectiva do *Nurturing Care*.

## Método

### Delineamento do estudo

Trata-se de um estudo com abordagem qualitativa, na perspectiva da hermenêutica gadameriana^(15)^, ancorada em um modo de conceber e estabelecer relações com o outro. Essa abordagem baseia-se em um processo em que o diálogo é o *locus* da comunicação^(16)^, enfatizando a compreensão das situações e das experiências dos participantes. Esta pesquisa centra-se nas percepções parentais maternas sobre o cuidado de crianças menores de um ano de idade no contexto domiciliar, voltadas para o desenvolvimento infantil, e fundamenta-se na abordagem conceitual do *Nurturing Care* ^(2)^.

O estudo seguiu as diretrizes do *Consolidated Criteria for Reporting Qualitative Research* (COREQ)^(17)^.

### Local

O estudo foi realizado no município de Itaú de Minas, em Minas Gerais, Brasil, na área de abrangência das unidades com Estratégia Saúde da Família (ESF). Trata-se de um município de pequeno porte, com população estimada de 16.286 habitantes^(18)^, e que conta ao todo com cinco unidades de saúde, todas vinculadas à ESF. As cinco unidades abarcam a área urbana, sendo que uma delas também atende a zona rural do município, que não foi incluída no público-alvo da pesquisa.

### Período

O período de realização da pesquisa no município foi de 02 de maio a 31 de julho de 2023.

### Critérios de seleção

Os critérios de inclusão foram: mulheres acima de 18 anos de idade, que tiveram gestação de risco habitual, cadastradas e em acompanhamento nas unidades ESF, residentes em domicílio e com crianças de até um ano de idade. O critério de exclusão foi: crianças com necessidades especiais. As informações foram obtidas em prontuários das unidades de saúde. Foram consideradas perdas as participantes que não puderam ser encontradas após três tentativas de realização da visita domiciliar (VD).

### Participantes

As cinco unidades ESF contavam com um universo de 114 mulheres com crianças menores de um ano de idade. Foi realizada a verificação dos critérios de inclusão e exclusão, e também um levantamento da distribuição entre as unidades, identificando o seguimento em puericultura para crianças com menos de 12 meses. Ao final, 41 mulheres atenderam aos critérios. Foram incluídas as crianças menores de um ano de idade nascidas entre 02/05/2022 e 30/04/2023. Com relação às unidades de saúde, a distribuição final foi de 10 participantes da área da ESF-1, cinco da ESF-2, cinco da ESF-3, quatro da ESF-4 e três da ESF-5.

### Instrumentos utilizados para a coleta das informações

A coleta das informações foi realizada a partir de entrevistas semiestruturadas, contendo a caracterização das mães (idade, escolaridade, presença do pai da criança ou companheiro, renda familiar e trabalho materno) e das crianças (idade e sexo), e a pergunta norteadora “*Como têm sido as rotinas de estímulo e aprendizagem para cuidar da/o nome da criança no dia a dia?“.*

### Coleta de dados

O contato inicial com as entrevistadas foi realizado nas unidades de saúde, com o objetivo de explicar o propósito da pesquisa, a forma de realização, e obter a aquiescência para a visita domiciliar e a coleta dos dados. Preliminarmente, foram conduzidas duas entrevistas piloto para verificar a adequação no roteiro e possíveis ajustes; no entanto, essas entrevistas foram posteriormente desconsideradas, por terem sido incluídas perguntas adicionais.

As entrevistas ocorreram de forma presencial e individualizada, por meio de VD, e foram gravadas em áudio, mediante assinatura do Termo de Consentimento Livre e Esclarecido. A pergunta norteadora foi utilizada para iniciar a entrevista, seguida por outras questões encadeadas, perfazendo o mapa diário de 24 horas de atividades realizadas com a criança, particularmente nos períodos matutino, vespertino e noturno. Essa abordagem visou estabelecer uma atitude hermenêutica na interação com as entrevistadas.

Partiu-se do pressuposto de que ilustrar a rotina da criança a partir das percepções maternas possibilita uma análise envolvendo o cuidado, os estímulos e a vigilância do desenvolvimento infantil no ambiente domiciliar. Esses aspectos orientaram a formulação de perguntas adicionais articuladas aos domínios do *Nurturing Care*. Cada entrevista teve uma duração média de 50 minutos, e foi conduzida pela primeira autora. Notas de campo não foram registradas.

A interrupção das entrevistas foi baseada na composição dos relatos das participantes, considerando peculiaridades, riqueza e heterogeneidade dos dados qualitativos^(19)^, relativos ao desenvolvimento infantil e à abordagem conceitual adotada, pertinentes ao objeto de estudo.

### Tratamento e análise dos dados

A análise dos dados foi pautada na análise temática, seguindo uma abordagem indutiva. O processo de análise, focado nas experiências compartilhadas pelas participantes durante as entrevistas, foi amparado por uma atitude hermenêutica, compreendendo os saberes e descobertas acerca do outro e com o outro, e em preceitos do cuidado da criança presentes na abordagem do *Nurturing Care*.

A análise temática seguiu os passos de familiarização inicial com os dados, leituras repetitivas do material empírico, geração de códigos, definição de temas e interpretação descritiva e qualitativa^(20)^.

A transcrição das entrevistas na íntegra foi realizada pela primeira autora, com a organização dos arquivos digitais individuais no programa *Microsoft Word*®. Tais arquivos foram posteriormente lançados no *softwareAtlas.ti*® para gerenciar os dados e codificações das informações coletadas.

A codificação realizada pelo *software Atlas.ti*® trouxe elementos que foram discutidos pelas autoras, que realizaram a validação das unidades temáticas e temas oriundos dos relatos das participantes, garantindo a confiabilidade do estudo. As participantes foram identificadas com a letra M (M1, M2, M3...) e as crianças com a letra C (C1, C2, C3...), seguida pela idade em meses.

## Aspectos éticos

O projeto de origem do estudo obteve parecer favorável de Comitê de Ética em Pesquisa, com Certificado de Apresentação para Apreciação Ética número 6.056.891.

## Resultados

Os resultados apresentam os relatos parentais maternos de 27 mulheres, cujas características estão descritas na Tabela 1.

**Tabela 1-:** Características das mães participantes e suas crianças. Itaú de Minas, MG, Brasil, 2023

Características sociodemográficas		n	%
Idade materna	18 a 25 anos	11	42
	26 a 35 anos	11	42
	36 anos ou mais	5	16
Idade das crianças	0-3 meses	3	11
	4-6 meses	9	33
	7-9 meses	9	33
	10-11 meses	6	23
Área geográfica de residência	Urbana	27	100
	Rural	0	0
Nível de escolaridade	Ensino médio completo	14	52
	Ensino médio incompleto	8	30
	Ensino fundamental incompleto	4	15
	Ensino universitário completo	1	3
Estado de relacionamento	Com companheiro	24	89
	Sem companheiro	3	11
O pai da criança reside na mesma casa	Sim	22	81
	Não	5	19
Trabalha fora de casa	Sim	6	22
	Não	21	78
Renda mensal	< 1 SM*	4	15
	1-2 SM*	20	74
	≥ 3 SM*	3	11

*SM = Salário-mínimo vigente no Brasil em 2023, no valor de R$1.320,00

Nota-se uma parcela de mães jovens, sendo a maior parte com ensino médio completo e dedicadas ao trabalho doméstico. A renda familiar relatada pela maioria estava abaixo de três salários mínimos (SM), considerando o valor relativo ao ano de 2023 (R$1.320,00)^(21)^. Neste contexto, metade das participantes afirmou não enfrentar dificuldades econômicas, e outras relataram algum tipo de necessidade financeira. Todos os pais ou companheiros tinham um trabalho externo.

As idades das crianças variaram de dois a 11 meses. Com relação ao pai residir na mesma casa, a maioria das mães moravam com os pais biológicos das crianças. 

Os resultados qualitativos foram organizados em temas alinhados aos domínios do *Nurturing Care* estudados. Na Figura 1 são apresentados os temas e as unidades temáticas correspondentes.

**Figura 1-:** Temas e unidades temáticas dos resultados. Ribeirão Preto, SP, Brasil, 2024

A unidade temática *Interação com a criança: atenção e diálogo* aborda relatos sobre como as mães interagem e conversam com as crianças. *Ela presta atenção em tudo que eu falo. Então, acho que é muito bom, ela vai aprender vendo, sentindo, experimentando* (M27 C27, 7 meses). *A conversa faz parte do desenvolvimento deles, como que vão entender o que estamos falando? Na prática, falamos com eles, mostrando as coisas e explicando o que é* (M25 C25, 5 meses). Por outro lado, não há identificação do efeito da fala materna no desenvolvimento infantil, justificando que a criança não entende e não se concentra no que está sendo conversado com ela. *Conversar depende da idade, não é? É bom para aprender, mas na idade que ela está acho que não tem muita efetividade, porque ela nem presta atenção* (M6 C6, 4 meses).

A unidade temática *Brincar com e estimular as crianças* envolve a rotina em si, a atenção e o brincar, pautados como elementos importantes para o desenvolvimento infantil. *Rotina primeiramente, tem que ter, senão não damos conta. Além da rotina, carinho, atenção, acho que não pode faltar nunca* (M4 C4, 8 meses). *Brincar mais com ele, mostrar mais as coisas. Mostrar o mundo para ele e ensiná-lo a viver aqui, a adaptar frente as tempestades da vida* (M13 C13, 7 meses). As participantes apontam tipos de brincadeiras realizadas com a criança e as percepções desta atividade para a interação parental. *Brinca muito comigo e meu marido de esconder. Então, ela mesma já se esconde. Conversamos muito com ela, fazemos cosquinha, brincar de falar através dos bichinhos dela* (M3 C3, 10 meses). Os relatos trazem exemplos de brincadeiras durante os cuidados com a criança, sendo relevantes também para a preocupação com possíveis acidentes domésticos. *Durante o banho ele brinca demais, bate a mãozinha na água, tem os brinquedos dele, o patinho, e fica jogando água em mim, faz uma festa* (M17 C17, 9 meses). *Toda hora quer ficar pegando a vasilha de shampoo, do condicionador, coloco um patinho de borracha também. Se bobear bebe até a água suja da banheira, tem que ficar de olho nele* (M24 C24, 8 meses).

A unidade temática *Observando o desenvolvimento infantil* traz a apreensão materna acerca do desenvolvimento pelos sinais que a criança demonstra, seus movimentos e sua relação com o ambiente. *Já sabe abrir e fechar a mão, se eu chamo ela responde e vem ao meu encontro, já sabe onde está o pé, a mão, o nariz* (M1 C1, 10 meses). *Olha para nós e mostra que ela está vendo o gatinho. Antes não tinha noção de nada, mas quando comecei a conversar com ela, mostrar o gatinho, por exemplo, já dá para ver que ela começou a reconhecer* (M6 C6, 4 meses). Outras situações foram apontadas como preocupantes por não ter atingido alguns marcos do desenvolvimento de acordo com a idade. *Ele não engatinha ainda, fica bem pouco no andador, em vista dos meus outros filhos ele é mais mole, fala “quê”, não aprendeu a pronunciar nada ainda, mas grita bastante* (M10 C10, 11 meses).

Na unidade temática *Cotidiano infantil e participação parental paterna* é tratada a função do cuidador parental paterno enquanto interação e inserção no cuidado. A atuação positiva é relatada por meio de brincadeiras e execução de algumas ações. *O pai é louco por ela, brinca demais, fica com ela enquanto tomo banho, troca fralda, dá mamá. A maior parte dos dias fica com ela à noite* (M1 C1, 10 meses). O cuidado paterno é comparado ao materno: *O pai dele é uma versão completamente diferente de mim. Ele é atencioso, calmo com os meninos* (M11 C11, 3 meses). O tempo parental paterno dispendido com a criança é relatado como restrito, por momentos denominado de ajuda à mãe. *Ele é muito prestativo, e quando ele chega às quatro da tarde, ele pega os meninos, dá banho no mais velho, fica brincando com o bebê enquanto faço a janta, troca de vez em quando* (M4 C4, 8 meses). *Participa quando ele está aqui. Ele fica olhando para eu fazer almoço, para fazer jantar* (M7 C7, 8 meses). A pouca atuação paterna, do ponto de vista materno, é associada ao pouco tempo com a criança, sendo percebido pelas mães como um dilema. *É do jeito dele. Chega ‘morto’, brinca só um pouco e logo já dá para mim* (M5 C5, 11 meses). *Ele fica com ela depois do serviço um pouco. Mas trocar fralda, dar banho, fazer dormir, sou eu* (M21 C21, 8 meses). *Passa o dia todo no serviço. Aí chega cansado e não tem muito contato* (M13 C13, 7 meses).

As entrevistas maternas trouxeram que 14 pais ou companheiros participavam dos cuidados com a criança e 13 não participavam. Das 27 mulheres, 25 tinham apoio de cuidadores não-parentais, sendo 20 de avós da criança, três de irmãos acima de 20 anos de idade, um de tia e um de babá. Quando o pai biológico não morava com a criança, a interação foi relatada por meio de visitas e de contribuição financeira. *Ele participa só financeiramente. Ele me liga e manda mensagem para mim perguntando se ela está bem. Só a vê por vídeo* (M12 C12, 9 meses).

A unidade temática *O passeio como promotor de experiências no ambiente proximal* aborda as oportunidades ofertadas às crianças fora de casa, com a utilização do passeio como modo de distração. *Ele gosta de sair, passear. Vamos na pracinha, ou quando vou buscar um dos irmãos na escolinha, aí levo ele junto* (M10 C10, 11 meses). *Passeio muito com ele pelas ruas, coloco ele no carrinho e ando com ele, já levei no parquinho, já levei para comer algodão doce na pracinha, ele adora* (M23 C23, 6 meses). Há relatos sobre dificuldades com este tipo de atividade, evitando levar a criança frequentemente para passear. *Ele não para um segundo e fica muito difícil para ficarmos correndo atrás dele, cansa demais. Aí, preferimos deixar ele solto aqui em casa mesmo* (M15 C15, 11 meses). Também foi apontado o receio acerca de doenças que podem ser adquiridas. *Não gosto de ficar saindo muito com ela, tenho medo de doença* (M20 C20, 6 meses).

A unidade temática *Oportunidades de interação social* enfatiza a convivência com irmãos e outras crianças. *O irmão brinca muito com ela, mexe com ela, faz cócegas, brinca de esconde-achou* (M21 C21, 8 meses). A interação com outras crianças também foi relatada pela convivência com a família estendida. *Eu levo na minha avó, lá é cheio de crianças, os nossos primos. Vou pelo menos uma vez na semana lá, ela fica só observando e rindo de tudo* (M6 C6, 4 meses). *Só quando eu o levo para passear fora de casa, nas avós, aí vê os primos mais pequenos* (M19 C19, 5 meses). O relacionamento na vizinhança e outros ambientes foi também mencionado, trazendo conexão com elementos de interação e estímulos. *Tem a filha da vizinha, direto vou lá conversar com ela e as duas pequeninhas ficam ‘conversando’ também* (M9 C9, 5 meses). *Se eu sair ali fora com ela, quer ver todo mundo, dá tchau, fica rindo, faz ‘joia’ com a mão. Brinca com o cachorrinho, passa a mão, puxa a patinha dele* (M12 C12, 9 meses).

A unidade temática *Leitura de histórias e suas dificuldades para a implementação na rotina* traz o modo de realização da leitura para as crianças e as percepções maternas sobre esta atividade. Com relação ao momento da leitura, as mães apontam realizar com a criança, lendo, conversando e mostrando figuras. *Ele tem os livrinhos dele, coloco ali no colchão e fico mostrando as figuras para ele, falando de animais, frutas, apontando para ele* (M10 C10, 11 meses). Há também situações de associação da leitura para a criança com valores religiosos. *Já contei histórias. Mas são mais da bíblia, fico falando muito sobre Deus, José. Converso bastante com ele* (M5 C5, 11 meses).

A existência de livros infantis no domicílio não garante a sua utilização, sendo apontadas dificuldades pela falta de interesse da criança. *Ontem mesmo eu tentei. Mas não presta atenção. Acho que ela ainda não entende* (M3 C3, 10 meses). *Tem vários livros espalhados pela casa. Ler, assim, é mais complicado, ela não para quieta para escutar* (M12 C12, 9 meses). Outras dificuldades emergiram, como a falta do hábito de leitura das mães e a percepção de que as crianças não entendem devido à sua faixa etária. *Não é um hábito meu. Então, não tenho essa iniciativa, e ela não fica muito tempo prestando atenção* (M1 C1, 10 meses). *Até em questão do livrinho, não temos, vou ver se compro para ficar mostrando as figuras. Mas acho que só a partir de um aninho, parece que não entende nada agora* (M7 C7, 8 meses).

A unidade temática *Percepções maternas sobre a educação infantilpara o desenvolvimento na primeira infância* traz aspectos sobre a inserção da criança na creche. Do total das participantes, 17 pretendiam colocar a criança na creche aos dois anos de idade, critério estabelecido pelo município do estudo. As percepções favoráveis indicam benefícios da educação infantil. *Ela terá contato com outras crianças, outras pessoas, e isso contribuirá para o desenvolvimento, os estudos dizem isso* (M6 C6, 4 meses). *Vai ajudar, não é? Porque tem criança para ela brincar, interagir. Vai saber desde pequena compartilhar o espaço, saber falar para comunicar o que quer* (M27 C27, 7 meses). *Lá dão mais atenção do que nós em casa. Lá as cuidadoras ficam por conta das crianças, cantam, brincam, tem o momento do cochilo, tudo organizado* (M10 C10, 11 meses). *Lá ensina muita coisa, não é? Conhece um monte de crianças, tem horário para comer, para dormir, tudo, e isso acaba me ajudando e também a ela, para seguir uma rotina fixa mesmo* (M16 C16, 3 meses).

Houve relatos sobre a não inserção das crianças em creches, apontando justificativas para a decisão. *Não pretendo porque eu não trabalho, não é? Aí ela não vai para creche, nem mais velha, só quando precisar ir para a escola mesmo* (M3 C3, 10 meses). *Não pretendo, tenho medo das doenças e também vejo tanta creche maltratando as crianças, fico com medo deles maltratarem ele* (M14 C14, 11 meses).

A unidade temática *Medidas utilizadas para consolar as crianças* retrata as formas para a cessação do choro e atendimento das necessidades infantis. *Para consolar eu pego no colo, mostro os brinquedos, levo na horta daqui de casa. Às vezes, coloco no carrinho e dou uma volta no quarteirão, para tentar distrair ela* (M7 C7, 8 meses). *Para consolar só colocando no colo, dando o peito mesmo* (M16 C16, 3 meses). Houve relato de utilizar medicação para acalmar a criança, o que chama a atenção para possíveis incertezas com suposição de situação de dor e uso indiscriminado de medicações em domicílio. *Medico ela com dipirona, muito colo, levo para passear aqui na rua, distraio ela com a televisão* (M12 C12, 9 meses).

A unidade temática *Hábitos diários no uso de telas* está relacionada à rotina para assistir programas, com destaque para a televisão. *Lá pelo meio dia e meia, assiste televisão junto comigo. Dou a mamadeira para ela e ela dorme. Acorda de tarde e dou banho nela, troco de roupinha e deixo a televisão ligada para ela se entreter* (M6 C6, 4 meses). *Assiste televisão praticamente a manhã toda e à tarde um pouco. Se eu tenho algo para fazer, não tem jeito, fica assistindo algo e eu vigiando de longe* (M10 C10, 11 meses). Houve apontamentos sobre as vantagens do uso de telas para a criança. *As telas hoje em dia acabam ajudando bastante a gente, não é?* (M1 C1, 10 meses). *A televisão acaba que ajuda um pouco, chama muito a atenção da criança e ensina muita coisa* (M26 C26, 4 meses). O uso do celular é apontado como forma de consolar, intermeado com brincadeiras. *Para consolar buscamos brincar com ela, fazer cosquinha, colocar desenho para ela no celular* (M6 C6, 4 meses).

A unidade temática *Estabelecendo limites no cotidiano da criança* traz as formas e o uso de restrições que são praticados em atividades com a criança. A palavra “não” de modo enfático e repetidas vezes é relatada como o recurso para atribuir limites, com a finalidade de a criança entender e aprender o que se espera dela. *Geralmente eu falo o tal temido “não”, a primeira vez de uma forma calma. Mas ela está na fase da insistência, vai lá e tenta fazer de novo. Aí, eu mudo o tom de voz, falo bem firme com ela e ela desiste, faz biquinho, mas não chora, e distraímos ela com outra coisa* (M1 C1, 10 meses). *Falo muito não para ela, tiro a mão da tomada e falo não, faço cara de séria, aí ela faz biquinho tentando convencer a deixar fazer. Aí, tiro ela de perto e distraio ela* (M18 C18, 9 meses). A mudança do tom de voz e expressões faciais são também relatadas, a fim de que a criança perceba que está fazendo algo errado. *Para colocar os limites falo firme com ela, faço cara feia ou de decepção. Acho que agora que ela tá começando a entender* (M6 C6, 4 meses)*. Faço cara feia, engrosso a voz e falo firme, que ela não pode, que vai machucar, que não é de neném* (M9 C9, 5 meses).

Outra abordagem foi o uso de castigo para colocar limites, como um modo de punir a criança em situação de desobediência. *Já até coloquei de castigo estes dias, fica querendo colocar os brinquedos nas tomadas. Já falei que não podia, mas não escutou. Aí, peguei ele, coloquei no berço e falei que estava de castigo* (M15 C15, 11 meses). O estabelecimento de limites é visto como ação difícil, justificada pelo não entendimento da criança por ser muito pequena. *Ele não costuma entender o que eu digo, então não coloco muito limite* (M13 C13, 7 meses). *Ainda não entende quase nada, só a retiro de perto ou tiro da mão dela quando é algo que não pode* (M26 C26, 4 meses).

A unidade temática *Expectativas para o futuro da criança* traz alguns desejos maternos para a vida da criança, ressaltando a preocupação com circunstâncias sociais atuais e a busca por proteção divina. *Não sabemos o dia de amanhã. Fico muito preocupada de ver a situação que está o mundo de hoje, não é? Como é que vai ser essa criança. Torço que Deus acompanhe, que dará tudo certo. Tornar uma pessoa bem maravilhosa, não é? Estudiosa. Mas só Deus sabe* (M2 C2, 6 meses). A educação na vida da criança é destacada como um item que resulta em boas perspectivas. *Eu quero que ela estude muito, não é? Estudar bastante. Quero que ela estude bastante. Acho que ela tem tudo para ser bem estudiosa e muito inteligente* (M9 C9, 5 meses). *Eu quero que ela estude muito e vá embora daqui, vai fazer a vida dela, vai ter as coisas dela* (M22 C22, 2 meses). Algumas características socioemocionais, articuladas à importância da educação, são ressaltadas como anseios para a vida da criança. *Eu quero que ele seja uma pessoa boa, que saiba se relacionar com todo mundo mesmo com as diferenças que existem, que ele possa também ser alguém na vida, que seja muito estudioso* (M15 C15, 11 meses). *Espero que ele seja determinado, sonhador, mas que lute para conseguir o que quer, que seja inteligente e que estude muito para garantir um emprego bom, ter suas coisas e quem sabe sua família, se ele quiser se casar* (M8 C8, 6 meses).

O conjunto dos resultados foi analisado e inter-relacionado com os domínios do *Nurturing Care*, destacados em cuidados responsivos, aprendizagem oportuna e segurança e proteção (Figura 2).

**Figura 2-:** Domínios do *Nurturing Care* relacionados aos aspectos do mapa diário da criança. Ribeirão Preto, SP, Brasil, 2024

Os resultados expressam componentes da rotina da criança a partir de percepções maternas, incluindo elementos que tanto qualificam o ambiente de cuidado quanto apresentam fragilidades e incertezas para o desenvolvimento infantil em contexto domiciliar e no seu entorno.

## Discussão

As práticas parentais maternas apreendidas envolveram a rotina do ambiente de cuidados da criança, com elementos sobre interagir e brincar com a criança, reconhecer o desenvolvimento infantil, identificar a participação paterna, o convívio com outras crianças, a leitura de livros, o uso de telas, a inserção na educação infantil, as medidas para consolo, o estabelecimento de limites e as expectativas futuras. Este conjunto de elementos traz desdobramentos para o desenvolvimento infantil e está inter-relacionado com os domínios do *Nurturing Care* em cuidados responsivos, aprendizagem oportuna, e segurança e proteção da criança em seu primeiro ano de vida.

Os relatos maternos trazem à tona alguns modos de interação parental, incluindo atenção para com a criança e observação se ela está se desenvolvendo de acordo com a idade. Durante a primeira infância, interações parentais com as crianças são uma base essencial para o desenvolvimento, principalmente para a memória, a flexibilidade e a criatividade^(2,22)^. A aprendizagem em casa, principalmente permeada por interações parentais, tem influência potente e positiva nos resultados do desenvolvimento na primeira infância, em comparação com outros períodos, devido à velocidade do desenvolvimento inicial do cérebro^(23)^. Os ambientes positivos podem trazer impactos para o desenvolvimento das crianças, com efeito também para a saúde mental parental^(24)^.

As práticas parentais maternas trouxeram que a convivência com outras crianças é acionada na própria família, com a vizinhança e amigos, e é relacionada às oportunidades de estímulo, referentes a brincar junto, despertar sorriso, observar, apontar e mostrar objetos, configurando interação e aprendizado. O engajamento parental com atividades lúdicas se mostra benéfico para o desenvolvimento infantil^(25)^. Um leque de estimulação é importante, abrangendo a estimulação sensorial, das potencialidades da criança e da interação adulto-criança^(26)^. A utilização de brincadeiras com utensílios domésticos, a exemplo de recipientes de plástico e papelão vazios, ajuda o aprendizado pela exposição às diferentes texturas, formas e cores, facilitando o envolvimento ativo das crianças^(27)^. As interações de “esconde-achou” contribuem para estimular o desenvolvimento motor, socioemocional, cognitivo e da linguagem, bem como podem reduzir os problemas de comportamento na primeira infância^(6,25)^. Outro aspecto a ressaltar é a importância dos conhecimentos sobre o desenvolvimento infantil pelos cuidadores parentais, de modo a observar e reconhecer as fases, marcos e demandas específicas para o progresso da criança^(28)^.

A leitura de livros de histórias infantis esteve presente nos relatos maternos como atividade positiva, mas também houve dúvidas sobre a faixa etária e o pouco interesse da criança pequena. Neste aspecto, outros estudos identificaram que a posse de livros infantis teve maior grau de associação com interações parentais com as crianças e incremento da linguagem, em comparação com a rotina de brincar e cantar^(29-30)^. Em contraponto, um estudo encontrou que os cuidadores parentais raramente realizavam leitura de histórias com as crianças^(31)^.

No presente estudo, levar as crianças para passear foi considerado como um momento de espairecer e ofertar outras experiências infantis de apreciação dos ambientes. Alguns relatos expressam preocupações com a aquisição de doenças, com o comportamento da criança, e questões que permeiam a amamentação em público. As brincadeiras ao ar livre têm sido relacionadas às oportunidades para as crianças desenvolverem independência, autodeterminação e habilidades físicas^(32)^, além dos benefícios sociais, emocionais e cognitivos, incluindo elementos positivamente desafiadores^(33)^.

O uso de telas foi relacionado às possibilidades de as mães realizarem atividades domésticas, justificando a televisão ou o uso do celular como modo de entreter a criança. Chama a atenção o uso de celulares por crianças menores de um ano de idade, o que requer atuação junto aos cuidadores parentais com aconselhamento sobre os danos possíveis ao desenvolvimento infantil, buscando o engajamento parental e profissional quanto ao uso apropriado de telas para promover o bem-estar digital. Estudos demonstraram que a exposição excessiva aos meios digitais foi associada a problemas de desenvolvimento e comportamento em crianças pequenas^(34-35)^. Outro estudo enfatizou que as crianças que utilizaram menos tempo de telas interagiram mais com os cuidadores parentais^(29)^.

O colo e a distração no ambiente de cuidado da criança foram as práticas mencionadas para o seu consolo, como uma forma de estimular outros sentidos e acalmá-la. Outras maneiras para tirar o foco do choro foram brincadeiras, passeios, amamentação/alimentação, buscando distrações para evitar incômodos. Aspecto semelhante é apontado em estudo com cuidadores parentais que utilizavam práticas de distração para acalmar e evitar longos períodos de choro infantil^(36)^.

Nas práticas maternas relatadas, o estabelecimento de limites foi visto como um ensinamento de condutas, por vezes como uma forma de repreensão às atitudes das crianças. Ainda, houve considerações acerca da idade da criança, apontando certa referência ao processo de desenvolvimento infantil. Em outro estudo^(37)^, os cuidadores parentais indicaram não estar preparados para lidar com comportamentos desafiadores, a exemplo de acessos de raiva, e relataram utilizar práticas como gritos e palavrões. As experiências adversas na infância são consideradas interligadas às atitudes e comportamentos parentais negativos, levando a mais vulnerabilidades desenvolvimentais quando há atitudes parentais menos carinhosas e mais severas^(38)^. Assim, cabe ressaltar a importância de incluir experiências infantis protetoras, com propostas para diminuir atitudes hostis, agressivas e coercitivas, que são os principais preditores da violência familiar, e incrementar mudanças positivas na parentalidade^(39)^. Em complemento, ao tratar das percepções parentais, a literatura aponta a relevância de distinguir práticas parentais (indutivas, coercitivas), estilos parentais (autoritário, indulgente e negligente) e habilidades sociais educativas parentais (expressões de afeto, atenção, opiniões e direitos, e redução do uso de punições), que ampliam a reflexão sobre o desenvolvimento de intervenções por profissionais de diferentes áreas que trabalham com o cuidado de crianças^(40)^.

Os resultados também expressam a necessidade da prevenção de acidentes em domicílio no primeiro ano de vida, sendo importante integrá-la à interação, diálogo e estabelecimento de limites. Crianças pequenas são mais vulneráveis a lesões domésticas por estarem em processo de aquisição de habilidades físicas e sociais, o que requer oferta de aprendizagem segura e estimulante na implementação de medidas preventivas em casa^(41)^. Ademais, é importante considerar os fatores parentais nas injúrias não intencionais, como fragilidades na saúde mental parental, baixo envolvimento paterno nos cuidados infantis, agregado familiar monoparental, ambiente doméstico desorganizado, entre outros^(42)^.

A inserção da criança na educação infantil trouxe apontamentos maternos sobre as vantagens e desvantagens de uma rotina que pode proporcionar a aprendizagem e o convívio para um adequado desenvolvimento da criança. Houve também dúvidas, incertezas e medos quanto à qualidade do ambiente e atuação dos educadores. Os relatos maternos expressaram que a educação e o estudo são formas de obter sucesso na vida, aliados à felicidade e escolhas de bons caminhos. Houve um enfoque em valores religiosos como uma forma de acreditar que as crianças estarão resguardadas e no caminho certo para terem um futuro notável, com base nestas crenças.

Grande parte das participantes informou renda familiar menor do que três salários mínimos, e quando abordam expectativas para o futuro de suas crianças manifestam preocupações com as circunstâncias sociais em que vivem. Um estudo aponta que famílias que dependem fortemente de cuidadores não-parentais, como avós ou outras pessoas, trazem preocupações com a qualidade e o acesso da educação infantil^(43)^. Intervenções preventivas no âmbito dos cuidados primários em saúde da criança com acesso universal e elevado envolvimento indicam um impacto na redução de disparidades e no entendimento dos mecanismos pelos quais a pobreza pode criar barreiras à saúde relacional entre mães, pais e filhos^(44)^. Cabe destacar que as questões que envolvem as tomadas de decisão quanto aos cuidados alternativos exercidos por diferentes pessoas e instituições podem incidir positivamente ou negativamente no desenvolvimento nos primeiros anos de vida^(45)^.

No presente estudo, o conjunto dos resultados abordando as práticas parentais maternas trouxe elementos qualitativos para o desenvolvimento infantil, os quais estão inter-relacionados com os domínios do *Nurturing Care*, particularmente cuidados responsivos, aprendizagem oportuna, e segurança e proteção da criança, aqui focados em seu primeiro ano de vida. Reforça-se que tais domínios necessitam ser incrementados nos serviços de saúde, buscando garantir atenção de alta qualidade, informada por valores voltados às pessoas e que sejam equitativos, resilientes e eficientes^(46)^. Portanto, nos serviços de saúde, as intervenções parentais relacionadas à prevenção de agravos e promoção do desenvolvimento infantil são importantes nos primeiros três anos de vida^(6,47-48)^. Programas com ênfase em ajudar a lidar com desvantagens iniciais são relevantes para proporcionar estimulação responsiva e oportunidades de aprendizagem adequada^(49)^. Há também que serem repensadas as estratégias no sistema de cuidados primários para lidar com barreiras estruturais, desafios organizacionais e restrições financeiras^(50)^.

Este estudo apresenta implicações quanto ao cuidado de enfermagem em saúde da criança na APS, pelos resultados sobre as práticas parentais em domicílio, com destaque para o desenvolvimento de crianças menores de um ano de idade, fase em que muitas mudanças ocorrem e o cérebro em desenvolvimento é mais sensível às experiências e ao ambiente.

O cuidado de enfermagem em saúde da criança, em muitos momentos, há que identificar, lidar e intervir nas situações que englobam: (i) cuidados parentais responsivos, para incrementar conversas, atenção e carinho com a criança, analisar a rotina de cuidados, observar marcos do desenvolvimento infantil, incentivar a proatividade paterna, e identificar preocupações parentais de desenvolvimento; (ii) aprendizagem desde o início da vida apropriada a cada fase, para estimular por meio de brincadeiras, leituras, cantigas e passeios, e proporcionar contato com outras crianças; e (iii) segurança e proteção infantil, para equilibrar o consolo da criança e o estabelecimento de limites, prevenir acidentes domésticos e conhecer os benefícios da educação infantil qualificada. Portanto, apoiar cuidados responsivos e aprendizagem apropriada à idade, assim como intervir a favor da segurança e proteção da criança, configuram uma atuação profissional promissora e qualificada.

Na presente pesquisa, a atenção às situações de um mapa diário contribui para identificar as vulnerabilidades e atuar para reduzi-las, com base nos domínios do *Nurturing Care*. Assim, as intervenções oportunas, criativas e promotoras da saúde e do desenvolvimento infantil precisam ser intrínsecas ao cuidado de enfermagem em saúde da criança, em parcerias e corresponsabilidade parental para intervir em fragilidades e ampliar um apropriado ambiente de cuidado e desenvolvimento integral da criança. Os elementos qualitativos aqui analisados também reforçam a necessidade de fortalecer a continuidade do cuidado, inerente à atenção em puericultura, em visita domiciliar, no pré-natal e na gestão do cuidado infantil, que configuram importantes espaços do cuidado de enfermagem em saúde da criança na APS.

As limitações do estudo referem-se à realização das entrevistas apenas com as mães e foco no primeiro ano de vida das crianças, centradas em especificidades da realidade de um município de pequeno porte com unidades de ESF. Embora os domínios do *Nurturing Care* relacionados à boa saúde e nutrição adequada não tenham sido abordados, os resultados expressaram a relevância dos domínios dos cuidados responsivos, aprendizagem oportuna, segurança e proteção no primeiro ano de vida, destacando potencialidades e vulnerabilidades do desenvolvimento infantil no contexto domiciliar. Outras investigações são importantes a respeito de percepções de outros cuidadores parentais e não parentais, outras faixas etárias e adversidades ao desenvolvimento na primeira infância.

## Conclusão

As práticas parentais maternas foram analisadas com ênfase na percepção da rotina diária da criança voltada ao desenvolvimento infantil, trazendo qualidades e fragilidades do seu cotidiano. Ainda, houve ampliação da compreensão do ambiente de cuidado e interação, que oferece oportunidades de aprendizado e restrições no cotidiano, sugerindo expor as crianças às vulnerabilidades em momentos circunstanciais vitais para as suas necessidades de desenvolvimento desde tenra idade.

Os domínios do *Nurturing Care* referentes aos cuidados responsivos, aprendizagem oportuna, e segurança e proteção são muito importantes para a criança em seu primeiro ano de vida, indicando experiências de interação, diferentes momentos e ações no ambiente de cuidado domiciliar. O foco no primeiro ano de vida propicia a compreensão da dinamicidade do desenvolvimento infantil, apontando novas formas de perceber e conduzir os cuidados parentais. Assim, esses domínios possibilitam a identificação de circunstâncias que denotam vulnerabilidades e potencialidades do cuidado, a partir do seu caráter contingencial, porque implica lidar com a eventualidade, a incerteza e as diferenças dos acontecimentos vinculados às experiências.

A abordagem do *Nurturing Care* oferece domínios de análise no campo da APS, reafirmando a relevância de considerar a responsividade parental no cuidado da criança e de apreender sua rotina diária em aspectos substanciais ao desenvolvimento pleno e ao cuidado integral, sugerindo uma estratégia promotora do cuidado de enfermagem em saúde da criança.

# Translation: Parentalidad y estímulo al desarrollo infantil en el hogar: perspectivas del *Nurturing Care* *

**DOI:** 10.1590/1518-8345.7554.4610

**Authors:** Jeniffer Stephanie Marques Hilário, Letícia Pancieri, Regina Aparecida Garcia de Lima, Elsa Maria Oliveira Pinheiro de Melo, Débora Falleiros de Mello

**Affiliations:** 1 Universidade de São Paulo, Escola de Enfermagem de Ribeirão Preto, Centro Colaborador de la OPS/OMS para el Desarrollo de la Investigación en Enfermería, Ribeirão Preto, SP, Brasil.; 2 Becaria del Conselho Nacional de Desenvolvimento Científico e Tecnológico (CNPq), Brasil.; 3 Universidade de Aveiro, Faculdade de Ciências da Saúde, Aveiro, Portugal.

**Keywords:** Niño, Desarrollo Infantil, Cuidado Infantil, Responsabilidad Parental, Enfermería, Atención Primaria de Salud

## Abstract

analizar prácticas parentales maternas orientadas al desarrollo de niños menores de un año en contexto domiciliario, desde la perspectiva del *Nurturing Care*.

estudio cualitativo basado en la hermenéutica filosófica y enfoque conceptual del *Nurturing Care*. Se realizaron entrevistas semiestructuradas en profundidad con 27 madres y análisis temático inductivo.

las prácticas parentales maternas incluyeron interacción y juego con el niño, convivencia con otros niños, introducción del uso de pantallas desde edad temprana, relativa participación paterna y lectura ocasional de libros. Establecimiento de límites, inserción en la educación infantil y percepción del desarrollo infantil se destacaron por sus incertidumbres. El conjunto de resultados indicó interrelación parcial con los dominios del *Nurturing Care* en cuanto a cuidados responsivos, aprendizaje oportuno, y seguridad y protección infantil.

las prácticas parentales maternas, desde el enfoque del *Nurturing Care*, reflejaron el entorno de cuidado del niño, señalando fortalezas y vulnerabilidades para promover el desarrollo infantil en contexto domiciliario. Los dominios del *Nurturing Care* son útiles para el cuidado infantil en la Atención Primaria de Salud, reafirmando la importancia de la responsividad parental y la rutina diaria del niño para su desarrollo pleno, sugiriendo una estrategia promotora del cuidado de enfermería en salud infantil.

## Destacados

**(1)** La rutina diaria en el hogar retrata prácticas parentales orientadas al desarrollo infantil. **(2)** Los relatos maternos expresan cualidades y fragilidades del entorno de cuidado infantil. **(3)** Un entorno de cuidado e interacción brinda oportunidades de aprendizaje infantil. **(4)** Las restricciones en la vida cotidiana exponen a los niños a vulnerabilidades en su desarrollo. **(5)** El enfoque del *Nurturing Care* ofrece dominios de análisis sobre el cuidado infantil en el hogar.

## Introducción

En la primera infancia, período que abarca los primeros seis años de vida, ocurre un intenso desarrollo cerebral, influenciado por la formación de estructuras que reciben múltiples estímulos de las interacciones entre los cuidadores y el niño, así como de los entornos en los que se inserta^(1)^, siendo de vital importancia un cuidado adecuado y una variedad de experiencias^(2)^.

Brindar condiciones favorables para el desarrollo infantil es más eficaz y menos costoso que intentar mitigar posteriormente los efectos de las adversidades^(3)^. En términos de políticas públicas, se han dirigido esfuerzos para promover el desarrollo pleno de todos los niños^(4)^. La Agenda 2030, compuesta por los 17 Objetivos de Desarrollo Sostenible (ODS), incluye la promoción del desarrollo infantil, resaltando la necesidad del acceso universal a los servicios de salud para garantizar una atención adecuada en la infancia^(5)^.

En la primera infancia, el alcance de los ODS está vinculado a la garantía del *Nurturing Care* para los niños^(2)^. El enfoque del *Nurturing Care* enfatiza el desarrollo pleno en la primera infancia y se compone de los siguientes dominios: buena salud, nutrición adecuada, cuidados responsivos, aprendizaje desde una edad temprana, y seguridad y protección del niño^(2)^. Sin embargo, estudios han indicado que este enfoque se ha centrado más en los componentes de salud y nutrición, con menor atención al aprendizaje temprano, los cuidados responsivos, y la seguridad y protección^(6-8)^, especialmente en el cuidado de niños pequeños en países de ingresos bajos y medianos. Las evidencias científicas destacan la importancia de la vigilancia del desarrollo infantil y el monitoreo de sus hitos, identificando condiciones adversas y analizando sus consecuencias a corto y largo plazo^(9-10)^.

Se destaca la necesidad de priorizar el desarrollo en niños menores de un año, dado que existe una probabilidad significativa de que presenten problemas en su desarrollo durante los primeros años de vida^(11)^. Asimismo, las intervenciones destinadas a mejorar los resultados orientados a los cuidadores parentales y a apoyar el desarrollo infantil requieren avances^(12)^. Además, el cuidado de enfermería en salud infantil tiene un gran potencial para la evaluación de la salud y el desarrollo infantil, así como para la implementación de intervenciones oportunas, considerándose elementos esenciales en la atención primaria de salud y aspectos fundamentales dentro de las competencias clínicas en este ámbito^(13-14)^.

Ante los desafíos contemporáneos presentes particularmente en países de ingresos bajos y medianos, incluido Brasil, existe una relevancia científica en centrarse en las prácticas parentales dirigidas a los niños en su primer año de vida, destacándose el desarrollo infantil en términos de cuidados responsivos, aprendizaje oportuno, y seguridad y protección a partir de percepciones maternas, aspecto de interés en esta investigación. En este sentido, se busca comprender las prácticas en el entorno del cuidado domiciliario, las potencialidades del *Nurturing Care* y sus implicaciones para el cuidado de enfermería en salud infantil en el ámbito de la Atención Primaria de Salud (APS). Así, el objetivo del estudio es analizar prácticas parentales maternas orientadas al desarrollo de niños menores de un año en contexto domiciliario, desde la perspectiva del *Nurturing Care*.

## Método

### Diseño del estudio

Se trata de un estudio con un enfoque cualitativo, desde la perspectiva de la hermenéutica gadameriana^(15)^, basada en una forma de concebir y establecer relaciones con el otro. Este enfoque se fundamenta en un proceso donde el diálogo es el *locus* de la comunicación^(16)^, enfatizando la comprensión de las situaciones y experiencias de los participantes. Esta investigación se centra en las percepciones parentales maternas sobre el cuidado de niños menores de un año en el contexto domiciliario, con énfasis en el desarrollo infantil, y se basa en el enfoque conceptual del *Nurturing Care* ^(2)^.

El estudio siguió las directrices del *Consolidated Criteria for Reporting Qualitative Research* (COREQ)^(17)^.

### Lugar

El estudio se llevó a cabo en el municipio de Itaú de Minas, en Minas Gerais, Brasil, en el área de cobertura de las unidades con la Estrategia de Salud de la Familia (ESF). Se trata de un municipio de pequeño porte, con una población estimada de 16.286 habitantes^(18)^, que cuenta con un total de cinco unidades de salud, todas vinculadas a la ESF. Las cinco unidades abarcan la zona urbana, y una de ellas también atiende la zona rural del municipio, que no fue incluida en el público objetivo de la investigación.

### Período

El período de realización de la investigación en el municipio fue del 2 de mayo al 31 de julio de 2023.

### Criterios de selección

Los criterios de inclusión fueron: mujeres mayores de 18 años, con gestación de bajo riesgo, registradas y en seguimiento en las unidades de la ESF, residentes en domicilio y con niños de hasta un año de edad. El criterio de exclusión fue: niños con necesidades especiales. La información fue obtenida de los historiales médicos de las unidades de salud. Se consideraron pérdidas aquellas participantes que no pudieron ser contactadas después de tres intentos de visita domiciliaria (VD).

### Participantes

Las cinco unidades de la ESF contaban con un universo de 114 mujeres con niños menores de un año. Se realizó la verificación de los criterios de inclusión y exclusión, además de un relevamiento de la distribución entre las unidades, identificando el seguimiento en puericultura para niños menores de 12 meses. Al final, 41 mujeres cumplieron los criterios. Se incluyeron niños menores de un año nacidos entre el 02/05/2022 y el 30/04/2023. En cuanto a las unidades de salud, la distribución final fue de 10 participantes en el área de la ESF-1, cinco en la ESF-2, cinco en la ESF-3, cuatro en la ESF-4 y tres en la ESF-5.

### Instrumentos utilizados para la recolección de información

La recolección de información se realizó mediante entrevistas semiestructuradas, que incluían la caracterización de las madres (edad, nivel de escolaridad, presencia del padre del niño o de una pareja, ingresos familiares y trabajo materno) y de los niños (edad y sexo), además de la pregunta orientadora: “¿*Cómo han sido las rutinas de estímulo y aprendizaje para cuidar de nombre del niño/a en el día a día*?”*.*

### Recolección de datos

El contacto inicial con las entrevistadas se llevó a cabo en las unidades de salud, con el objetivo de explicar el propósito de la investigación, la forma de realización y obtener su consentimiento para la visita domiciliaria y la recolección de datos. Preliminarmente, se realizaron dos entrevistas piloto para verificar la adecuación del guion y posibles ajustes; sin embargo, estas entrevistas fueron posteriormente descartadas, ya que se incluyeron preguntas adicionales.

Las entrevistas se realizaron de forma presencial e individual, mediante VD, y fueron grabadas en audio tras la firma del Formulario de Consentimiento Libre e Informado. Se utilizó la pregunta orientadora para iniciar la entrevista, seguida de otras cuestiones encadenadas, formando un mapa diario de 24 horas con las actividades realizadas con el niño, particularmente en los períodos de la mañana, la tarde y la noche. Este enfoque tuvo como objetivo establecer una actitud hermenéutica en la interacción con las entrevistadas.

Se partió del supuesto de que ilustrar la rutina del niño a partir de las percepciones maternas permite un análisis del cuidado, los estímulos y la vigilancia del desarrollo infantil en el entorno domiciliario. Estos aspectos guiaron la formulación de preguntas adicionales vinculadas a los dominios del *Nurturing Care*. Cada entrevista tuvo una duración promedio de 50 minutos y fue conducida por la primera autora. No se registraron notas de campo.

La interrupción de las entrevistas se basó en la composición de los relatos de las participantes, considerando las particularidades, la riqueza y la heterogeneidad de los datos cualitativos^(19)^, en relación con el desarrollo infantil y el enfoque conceptual adoptado, pertinentes al objeto de estudio.

### Procesamiento y análisis de los datos

El análisis de los datos se basó en el análisis temático, siguiendo un enfoque inductivo. El proceso analítico, centrado en las experiencias compartidas por las participantes durante las entrevistas, se sustentó en una actitud hermenéutica, buscando comprender los conocimientos y descubrimientos sobre el otro y con el otro, así como en los principios del cuidado infantil presentes en el enfoque del *Nurturing Care*.

El análisis temático siguió los pasos de familiarización inicial con los datos, lecturas repetitivas del material empírico, generación de códigos, definición de temas e interpretación descriptiva y cualitativa^(20)^.

La transcripción íntegra de las entrevistas fue realizada por la primera autora, organizando los archivos digitales individuales en el programa *Microsoft Word*®. Posteriormente, estos archivos fueron ingresados en el *softwareAtlas.ti*® para gestionar los datos y codificar la información recolectada.

La codificación realizada por el *software Atlas.ti*® proporcionó elementos que fueron discutidos por las autoras, quienes validaron las unidades temáticas y los temas surgidos de los relatos de las participantes, garantizando la confiabilidad del estudio. Las participantes fueron identificadas con la letra M (M1, M2, M3...) y los niños con la letra C (C1, C2, C3...), seguida de la edad en meses.

## Aspectos éticos

El proyecto original del estudio obtuvo un dictamen favorable del Comité de Ética en Investigación, con el Certificado de Presentación para Apreciación Ética número 6.056.891.

## Resultados

Los resultados presentan los relatos parentales maternos de 27 mujeres, cuyas características se describen en la Tabla 1.

**Tabla 1-:** Características de las madres participantes y sus niños. Itaú de Minas, MG, Brasil, 2023

Características sociodemográficas		n	%
Edad materna	18 a 25 años	11	42
	26 a 35 años	11	42
	36 años o más	5	16
Edad de los niños	0-3 meses	3	11
	4-6 meses	9	33
	7-9 meses	9	33
	10-11 meses	6	23
Área geográfica de residencia	Urbana	27	100
	Rural	0	0
Nivel de escolaridad	Educación secundaria completa	14	52
	Educación secundaria incompleta	8	30
	Educación primaria incompleta	4	15
	Educación superior completa	1	3
Estado de relación	Con pareja	24	89
	Sin pareja	3	11
El padre del niño reside en la misma casa	Sí	22	81
	No	5	19
Trabaja fuera de casa	Sí	6	22
	No	21	78
Ingreso mensual	< 1 SM*	4	15
	1-2 SM*	20	74
	≥ 3 SM*	3	11

*SM = Salario-mínimo vigente en Brasil en 2023, con un valor de R$1.320,00

Se observa una proporción de madres jóvenes, gran parte con educación secundaria completa y dedicadas al trabajo doméstico. La mayoría reportó ingresos familiares inferiores a tres salarios mínimos (SM), considerando el valor del año 2023 (R$1.320,00)^(21)^. En este contexto, la mitad de las participantes afirmó no enfrentar dificultades económicas, mientras que otras mencionaron algún tipo de necesidad financiera. Todos los padres o compañeros tenían un trabajo fuera del hogar.

Las edades de los niños variaron entre dos y 11 meses. En cuanto a la convivencia con el padre, la mayoría de las madres vivía con los padres biológicos de los niños. 

Los resultados cualitativos se organizaron en temas alineados con los dominios del *Nurturing Care* estudiados. En la Figura 1 se presentan los temas y sus respectivas unidades temáticas.

**Figura 1-:** Temas y unidades temáticas de los resultados. Ribeirão Preto, SP, Brasil, 2024

La unidad temática *Interacción con el niño: atención y diálogo* aborda relatos sobre cómo las madres interactúan y conversan con sus hijos. *Ella presta atención a todo lo que digo. Entonces, creo que es muy bueno, aprenderá viendo, sintiendo, experimentando* (M27 C27, 7 meses). *La conversación es parte de su desarrollo, ¿cómo van a entender lo que decimos? En la práctica, les hablamos, les mostramos cosas y les explicamos qué son* (M25 C25, 5 meses). Por otro lado, algunas madres no identifican el efecto del habla materna en el desarrollo infantil, justificando que el niño no entiende ni se concentra en lo que se le dice. *Hablar depende de la edad, ¿no? Es bueno para aprender, pero a la edad que tiene, creo que no es muy efectivo, porque ni siquiera presta atención* (M6 C6, 4 meses).

La unidad temática *Juego y estimulación de los niños* abarca la rutina, la atención y el juego como elementos fundamentales para el desarrollo infantil. *Primero, tiene que haber rutina, de lo contrario, no damos abasto. Además de la rutina, el cariño y la atención nunca pueden faltar* (M4 C4, 8 meses)*. Jugar más con él, mostrarle más cosas. Mostrarle el mundo y enseñarle a vivir aquí, a adaptarse a las tormentas de la vida* (M13 C13, 7 meses). Las participantes mencionan los tipos de juegos que realizan con sus hijos y cómo perciben esta actividad en la interacción parental. *Juega mucho al escondite conmigo y con mi marido. Ahora hasta se esconde sola. Hablamos mucho con ella, le hacemos cosquillas y jugamos a hablar a través de sus peluches* (M3 C3, 10 meses). Los relatos incluyen ejemplos de juegos durante el cuidado diario del niño, destacando también la preocupación por posibles accidentes domésticos. *Durante el baño, juega mucho, golpea el agua con sus manos, tiene sus juguetes, su patito, y me salpica agua, hace toda una fiesta* (M17 C17, 9 meses). *Todo el tiempo quiere agarrar los envases de champú y acondicionador. También le pongo un patito de goma. Si me descuido, hasta bebe el agua sucia de la bañera, hay que vigilarlo* (M24 C24, 8 meses).

La unidad temática *Observación del desarrollo infantil* refleja la percepción materna sobre el desarrollo a partir de las señales que el niño muestra, sus movimientos y su relación con el entorno. *Ya sabe abrir y cerrar la mano, si la llamo responde y viene hacia mí, ya reconoce dónde están el pie, la mano, la nariz* (M1 C1, 10 meses). *Nos mira y muestra que está viendo al gatito. Antes no tenía noción de nada, pero cuando empecé a hablarle y a mostrarle el gatito, por ejemplo, ya se nota que comenzó a reconocerlo* (M6 C6, 4 meses). Algunas situaciones fueron señaladas como preocupantes, ya que el niño no había alcanzado ciertos hitos del desarrollo según su edad. *Todavía no gatea, pasa muy poco tiempo en el andador; en comparación con mis otros hijos, es más flojito, dice ‘qué’, pero aún no ha aprendido a pronunciar nada, aunque grita bastante* (M10 C10, 11 meses).

La unidad temática *Vida cotidiana infantil y participación parental paterna* aborda el rol del cuidador parental paterno en términos de interacción e implicación en el cuidado. Se relatan experiencias positivas a través de juegos y la ejecución de algunas tareas. *El padre está loco por ella, juega mucho, se queda con ella mientras me baño, le cambia el pañal, le da el biberón. La mayoría de los días se queda con ella por la noche* (M1 C1, 10 meses). El cuidado paterno es comparado con el materno: *El padre es una versión completamente diferente de mí. Es atento, tranquilo con los niños* (M11 C11, 3 meses). El tiempo que los padres dedican a la crianza se describe como limitado y, en muchos casos, se enmarca como una ayuda a la madre. *Es muy servicial, y cuando llega a las cuatro de la tarde, se ocupa de los niños, baña al mayor, juega con el bebé mientras yo preparo la cena, y de vez en cuando cambia pañales* (M4 C4, 8 meses). *Participa cuando está aquí. Lo cuida mientras hago el almuerzo o la cena* (M7 C7, 8 meses). Desde la perspectiva materna, la baja participación paterna se asocia al poco tiempo que pasan con los niños, lo que genera un dilema para las madres. *Es a su manera. Llega agotado, juega un poco y enseguida me lo pasa a mí* (M5 C5, 11 meses). *Se queda con ella un rato después del trabajo. Pero cambiar pañales, bañar, hacerla dormir, todo lo hago yo* (M21 C21, 8 meses). *Pasa todo el día en el trabajo. Luego llega cansado y no tiene mucho contacto* (M13 C13, 7 meses).

Las entrevistas maternas revelaron que 14 padres o compañeros participaban en el cuidado del niño, mientras que 13 no lo hacían. De las 27 mujeres, 25 contaban con el apoyo de cuidadores no parentales, siendo 20 de los abuelos del niño, tres de hermanos mayores de 20 años, uno de una tía y uno de una niñera. Cuando el padre biológico no vivía con el niño, la interacción se describía a través de visitas y apoyo financiero. *Él solamente participa económicamente. Me llama y me manda mensajes preguntando si ella está bien. Solamente la ve por video* (M12 C12, 9 meses).

La unidad temática *El paseo como promotor de experiencias en el entorno próximo* aborda las oportunidades que se ofrecen a los niños fuera del hogar, utilizando el paseo como una forma de distracción. *Le gusta salir, pasear. Vamos a la placita o, cuando voy a buscar a uno de sus hermanos a la escuelita, lo llevo conmigo* (M10 C10, 11 meses). *Paseo mucho con él por las calles, lo pongo en el cochecito y camino con él, ya lo llevé al parque, a comer algodón de azúcar en la placita, le encanta* (M23 C23, 6 meses). También se mencionaron dificultades con este tipo de actividad, lo que hace que se evite llevar al niño de paseo con frecuencia. *No para ni un segundo y es muy difícil estar corriendo detrás de él, cansa demasiado. Así que preferimos dejarlo suelto aquí en casa* (M15 C15, 11 meses). Además, algunas madres expresaron preocupación por las enfermedades que el niño podría contraer al salir. *No me gusta salir mucho con ella, tengo miedo de que se enferme* (M20 C20, 6 meses).

La unidad temática *Oportunidades de interacción social* enfatiza la convivencia con hermanos y otras niñas y niños. *Su hermano juega mucho con ella, la provoca, le hace cosquillas, juegan al cucú* (M21 C21, 8 meses). La interacción con otros niños también se menciona en el contexto de la convivencia con la familia extensa*. La llevo a casa de mi abuela, allí hay muchos niños, nuestros primos. Voy al menos una vez por semana y ella se queda observando y riéndose de todo* (M6 C6, 4 meses). *Solamente cuando lo llevo a pasear fuera de casa, a la casa de sus abuelos, ahí ve a sus primos más pequeños* (M19 C19, 5 meses). El contacto con la vecindad y otros entornos también fue señalado, destacando la conexión con elementos de interacción y estímulo*. Está la hija de mi vecina, seguido voy a conversar con ella y las dos pequeñas también ‘conversan’ entre sí* (M9 C9, 5 meses). *Si salgo con ella a la calle, quiere ver a todo el mundo, saluda con la mano, se ríe, hace la señal de ‘ok’ con los dedos. Juega con el perrito, lo acaricia, le agarra la patita* (M12 C12, 9 meses).

La unidad temática *Lectura de cuentos y las dificultades para su implementación en la rutina* aborda la forma en que se realiza la lectura para los niños y las percepciones maternas sobre esta actividad. En cuanto al momento de la lectura, las madres mencionan que la realizan con el niño, leyendo, conversando y mostrando ilustraciones. *Tiene sus libritos, los pongo allá en el colchón y le voy mostrando las figuras, hablándole de los animales, las frutas, señalándoselas* (M10 C10, 11 meses). También se mencionan situaciones en las que la lectura para el niño está asociada con valores religiosos. *Le he contado historias, pero más de la Biblia. Le hablo mucho sobre Dios, sobre José. Converso bastante con él* (M5 C5, 11 meses).

La presencia de libros infantiles en el hogar no garantiza su uso, ya que se señalan dificultades relacionadas con la falta de interés del niño. *Ayer mismo lo intenté, pero no presta atención. Creo que todavía no entiende* (M3 C3, 10 meses). *Hay varios libros por la casa, pero leer así es más complicado, no se queda quieta para escuchar* (M12 C12, 9 meses). Otras dificultades mencionadas incluyen la falta de hábito de lectura por parte de las madres y la percepción de que los niños no comprenden debido a su edad. *No es un hábito mío, así que no tengo esa iniciativa, y ella no presta atención por mucho tiempo* (M1 C1, 10 meses). *Incluso con respecto a los libritos, no tenemos. Voy a ver si compro algunos para mostrarle las figuras. Pero creo que solamente a partir de un año, porque ahora parece que no entiende nada* (M7 C7, 8 meses).

La unidad temática *Percepciones maternas sobre la educación infantil para el desarrollo en la primera infancia* aborda aspectos relacionados con la inserción del niño en la guardería. De las participantes, 17 planeaban inscribir a sus hijos a los dos años de edad, criterio establecido por el municipio del estudio. Las percepciones favorables destacan los beneficios de la educación infantil. *Tendrá contacto con otros niños, otras personas, y eso contribuirá a su desarrollo, los estudios lo dicen* (M6 C6, 4 meses). *Va a ayudar, ¿no? Porque habrá niños con los que jugar e interactuar. Desde pequeña aprenderá a compartir el espacio y a comunicarse para expresar lo que quiere* (M27 C27, 7 meses). *Allí reciben más atención que en casa. Las cuidadoras se dedican completamente a los niños: cantan, juegan, tienen su momento de siesta, todo está organizado* (M10 C10, 11 meses). *Ahí les enseñan muchas cosas, ¿no? Conocen a muchos niños, tienen horarios para comer, dormir, todo. Eso me ayuda a mí y también a ella, para que siga una rutina estable* (M16 C16, 3 meses).

También hubo relatos sobre la decisión de no inscribir a los niños en guarderías, con diferentes justificaciones. *No lo tengo previsto porque no trabajo, ¿no? Así que no irá a la guardería, ni siquiera cuando sea más grande, solamente cuando tenga que ir a la escuela* (M3 C3, 10 meses). *No tengo intención, me da miedo que se enferme y además veo tantas noticias de guarderías donde maltratan a los niños, me da miedo que lo lastimen* (M14 C14, 11 meses).

La unidad temática *Medidas utilizadas para contener a los niños* retrata las formas empleadas para calmar el llanto y atender las necesidades infantiles. *Para contenerla, la cargo en brazos, le muestro los juguetes, la llevo al huerto de casa. A veces, la pongo en el cochecito y doy una vuelta por la cuadra para intentar distraerla* (M7 C7, 8 meses). *Para calmarlo, solamente en brazos y dándole el pecho* (M16 C16, 3 meses). También se reportó el uso de medicación para tranquilizar al niño, lo que genera preocupación por la posible incertidumbre ante un supuesto dolor y el uso indiscriminado de fármacos en el hogar. *Le doy dipirona, la cargo mucho en brazos, la llevo a pasear por la calle y la distraigo con la televisión* (M12 C12, 9 meses).

La unidad temática *Hábitos diarios en el uso de pantallas* está relacionada con la rutina de ver programas, con énfasis en la televisión. *Alrededor de las doce y media, ve televisión conmigo. Le doy el biberón y se duerme. Se despierta por la tarde, la baño, la cambio de ropa y dejo la televisión encendida para que se entretenga* (M6 C6, 4 meses). *Ve televisión prácticamente toda la mañana y un poco por la tarde. Si tengo algo que hacer, no hay otra opción, se queda mirando algo mientras yo la vigilo desde lejos* (M10 C10, 11 meses). Se mencionaron ventajas en el uso de pantallas para los niños. *Las pantallas hoy en día nos ayudan bastante, ¿no?* (M1 C1, 10 meses). *La televisión ayuda un poco, capta mucho la atención del niño y le enseña muchas cosas* (M26 C26, 4 meses). El uso del celular se menciona como una estrategia para calmar al niño, combinada con el juego. *Para calmarla, tratamos de jugar con ella, hacerle cosquillas y ponerle dibujos animados en el celular* (M6 C6, 4 meses).

La unidad temática *Establecimiento de límites en la vida cotidiana del niño* aborda las formas y el uso de restricciones que se practican en las actividades con la infancia. La palabra “no”, de manera enfática y repetida, se menciona como el principal recurso para establecer límites, con el propósito de que el niño comprenda y aprenda lo que se espera de él. *Generalmente digo el temido “no”, la primera vez con calma. Pero está en la fase de la insistencia, va y trata de hacerlo de nuevo. Entonces cambio el tono de voz, le hablo con firmeza y se rinde, hace un puchero, pero no llora, y la distraemos con otra cosa* (M1 C1, 10 meses). *Le digo “no” muchas veces, le aparto la mano del enchufe y le digo que no, me pongo seria, y ella hace un puchero tratando de convencerme de que la deje hacerlo. Entonces la aparto y la distraigo con otra cosa* (M18 C18, 9 meses). También se mencionan cambios en el tono de voz y expresiones faciales para que el niño perciba que está haciendo algo indebido. *Para poner límites, le hablo con firmeza, pongo cara de enojo o de decepción. Creo que ahora está empezando a entender* (M6 C6, 4 meses). *Pongo cara de enojo, bajo la voz y le hablo con firmeza, diciéndole que no puede, que se puede lastimar, que eso no es para bebés* (M9 C9, 5 meses).

Otro enfoque fue el uso del castigo para establecer límites, como una forma de sancionar al niño en situaciones de desobediencia. *Ya lo he puesto en penitencia estos días, sigue queriendo meter los juguetes en los enchufes. Ya le dije que no podía, pero no hizo caso. Entonces lo tomé, lo puse en la cuna y le dije que estaba en penitencia* (M15 C15, 11 meses). El establecimiento de límites se percibe como una acción difícil, justificándose en la falta de comprensión del niño debido a su corta edad. *No suele entender lo que le digo, así que no le pongo muchos límites* (M13 C13, 7 meses). *Todavía no entiende casi nada, solamente la aparto o le quito de la mano cuando es algo que no puede tener* (M26 C26, 4 meses).

La unidad temática *Expectativas para el futuro del niño* aborda algunos deseos maternos para la vida del niño, resaltando la preocupación por las circunstancias sociales actuales y la búsqueda de protección divina. *No sabemos qué pasará mañana. Me preocupa mucho ver la situación en la que está el mundo hoy, ¿no? Cómo será la vida de este niño. Espero que Dios lo acompañe y que todo salga bien. Que se convierta en una persona maravillosa, ¿no? Estudiosa. Pero solamente Dios sabe* (M2 C2, 6 meses). La educación en la vida del niño se destaca como un elemento clave para generar buenas perspectivas. *Quiero que estudie mucho, ¿no? Que estudie bastante. Creo que tiene todo para ser muy estudiosa e inteligente* (M9 C9, 5 meses). *Quiero que estudie mucho y se vaya de aquí, que haga su vida, que tenga sus cosas* (M22 C22, 2 meses). Algunas características socioemocionales, en conjunto con la importancia de la educación, son resaltadas como aspiraciones para el futuro del niño. *Quiero que sea una buena persona, que sepa relacionarse con los demás, incluso con las diferencias que existen, que también pueda ser alguien en la vida, que sea muy estudioso* (M15 C15, 11 meses). *Espero que sea determinado, soñador, pero que luche por lo que quiere, que sea inteligente y estudie mucho para asegurarse un buen empleo, tener sus cosas y, quién sabe, formar su propia familia si así lo desea* (M8 C8, 6 meses).

El conjunto de resultados fue analizado e interrelacionado con los dominios del *Nurturing Care*, destacándose en cuidados responsivos, aprendizaje oportuno y seguridad y protección (Figura 2).

**Figura 2-:** Dominios del *Nurturing Care* relacionados con aspectos del mapa diario del niño. Ribeirão Preto, SP, Brasil, 2024

Los resultados expresan componentes de la rutina del niño a partir de las percepciones maternas, incluyendo elementos que tanto cualifican el ambiente de cuidado como evidencian fragilidades e incertidumbres para el desarrollo infantil en el contexto del hogar y su entorno.

## Discusión

Las prácticas parentales maternas identificadas abarcaron la rutina del entorno de cuidado del niño, incluyendo aspectos como la interacción y el juego con el niño, el reconocimiento del desarrollo infantil, la identificación de la participación paterna, la convivencia con otros niños, la lectura de libros, el uso de pantallas, la inserción en la educación infantil, las medidas de contención, el establecimiento de límites y las expectativas para el futuro. Este conjunto de elementos tiene implicaciones para el desarrollo infantil y está interrelacionado con los dominios del *Nurturing Care*, en cuanto a cuidados responsivos, aprendizaje oportuno y seguridad y protección del niño durante su primer año de vida.

Los relatos maternos ponen de manifiesto algunas formas de interacción parental, incluyendo la atención hacia el niño y la observación de su desarrollo acorde a la edad. Durante la primera infancia, las interacciones parentales con los niños constituyen una base esencial para el desarrollo, especialmente en lo que respecta a la memoria, la flexibilidad y la creatividad^(2,22)^. El aprendizaje en el hogar, particularmente cuando está mediado por interacciones parentales, tiene una influencia potente y positiva en los resultados del desarrollo en la primera infancia, en comparación con otros períodos, debido a la rapidez del desarrollo cerebral en esta etapa^(23)^. Los entornos positivos pueden generar impactos en el desarrollo infantil, con efectos también en la salud mental de los padres^(24)^.

Las prácticas parentales maternas revelaron que la convivencia con otras niñas y niños se da dentro de la propia familia, con vecinos y amigos, y está vinculada a oportunidades de estímulo, como jugar juntos, provocar sonrisas, observar, señalar y mostrar objetos, configurando así interacciones y aprendizajes. El compromiso parental con actividades lúdicas se muestra beneficioso para el desarrollo infantil^(25)^. Un abanico de estímulos es fundamental, abarcando la estimulación sensorial, el desarrollo de las potencialidades del niño y la interacción adulto-niño^(26)^. El uso de juegos con utensilios domésticos, como recipientes de plástico y cartón vacíos, favorece el aprendizaje al exponer a los niños a diferentes texturas, formas y colores, facilitando su participación activa^(27)^. Las interacciones del “cucú” contribuyen a estimular el desarrollo motor, socioemocional, cognitivo y del lenguaje, además de poder reducir los problemas de comportamiento en la primera infancia^(6,25)^. Otro aspecto a destacar es la importancia de que los cuidadores parentales tengan conocimientos sobre el desarrollo infantil, permitiéndoles observar y reconocer las etapas, hitos y necesidades específicas para el progreso del niño^(28)^.

La lectura de libros de cuentos infantiles estuvo presente en los relatos maternos como una actividad positiva, aunque también surgieron dudas sobre la edad adecuada y el escaso interés de los niños pequeños. En este sentido, otros estudios identificaron que la posesión de libros infantiles tuvo una mayor asociación con las interacciones parentales y el desarrollo del lenguaje en comparación con la rutina de jugar y cantar^(29-30)^. Por otro lado, un estudio encontró que los cuidadores parentales rara vez realizaban la lectura de cuentos con los niños^(31)^.

En el presente estudio, llevar a los niños de paseo fue considerado un momento para despejarse y brindarles otras experiencias de exploración del entorno. Algunos relatos expresan preocupaciones sobre la adquisición de enfermedades, el comportamiento del niño, y cuestiones relacionadas con la lactancia materna en público. Las actividades al aire libre se han asociado con oportunidades para que los niños desarrollen independencia, autodeterminación y habilidades físicas^(32)^, además de beneficios sociales, emocionales y cognitivos, incluyendo elementos que representan desafíos positivos^(33)^.

El uso de pantallas se relacionó con la posibilidad de que las madres realizaran tareas domésticas, justificando la televisión o el uso del celular como una forma de entretener al niño. Llama la atención el uso de celulares por parte de niños menores de un año, lo que requiere la intervención de los cuidadores parentales mediante el asesoramiento sobre los posibles daños al desarrollo infantil, fomentando el compromiso tanto de los padres como de los profesionales en el uso adecuado de las pantallas para promover el bienestar digital. Los estudios han demostrado que la exposición excesiva a los medios digitales se asoció con problemas de desarrollo y comportamiento en niños pequeños^(34-35)^. Otro estudio destacó que los niños que pasaban menos tiempo frente a las pantallas interactuaban más con sus cuidadores parentales^(29)^.

El contacto físico y la distracción en el entorno de cuidado del niño fueron las prácticas mencionadas para su contención, como una forma de estimular otros sentidos y tranquilizarlo. Otras estrategias para desviar la atención del llanto incluyeron juegos, paseos, lactancia/alimentación, buscando distracciones para evitar molestias. Un aspecto similar fue señalado en un estudio con cuidadores parentales que empleaban prácticas de distracción para calmar al niño y evitar períodos prolongados de llanto infantil^(36)^.

En las prácticas maternas relatadas, el establecimiento de límites fue visto como una enseñanza de conductas y, en algunos casos, como una forma de reprender las actitudes de los niños. Además, se consideró la edad del niño, haciendo referencia al proceso de desarrollo infantil. En otro estudio^(37)^, los cuidadores parentales indicaron no estar preparados para manejar comportamientos desafiantes, como los episodios de rabietas, y mencionaron el uso de prácticas como gritos y palabras ofensivas. Las experiencias adversas en la infancia se consideran interconectadas con actitudes y comportamientos parentales negativos, lo que conlleva mayores vulnerabilidades en el desarrollo cuando las interacciones parentales son menos afectuosas y más severas^(38)^. Así, es fundamental resaltar la importancia de incluir experiencias infantiles protectoras, con estrategias para reducir actitudes hostiles, agresivas y coercitivas, que son los principales predictores de la violencia familiar, e impulsar cambios positivos en la parentalidad^(39)^. Además, al abordar las percepciones parentales, la literatura destaca la relevancia de diferenciar entre prácticas parentales (inductivas, coercitivas), estilos parentales (autoritario, indulgente y negligente) y habilidades sociales educativas parentales (expresión de afecto, atención, derechos y opiniones, y reducción del uso de castigos). Estas distinciones amplían la reflexión sobre el desarrollo de intervenciones por parte de profesionales de diversas áreas que trabajan en el cuidado infantil^(40)^.

Los resultados también expresan la necesidad de prevenir accidentes en el hogar durante el primer año de vida, destacando la importancia de integrarlos en la interacción, el diálogo y el establecimiento de límites. Los niños pequeños son más vulnerables a las lesiones domésticas debido a que están en proceso de adquisición de habilidades físicas y sociales, lo que requiere proporcionar un entorno de aprendizaje seguro y estimulante mediante la implementación de medidas preventivas en casa^(41)^. Además, es fundamental considerar los factores parentales en las lesiones no intencionales, como fragilidades en la salud mental de los cuidadores, baja participación paterna en el cuidado infantil, familias monoparentales, entorno doméstico desorganizado, entre otros^(42)^.

La inserción del niño en la educación infantil reflejó percepciones maternas sobre las ventajas y desventajas de una rutina que puede favorecer el aprendizaje y la socialización para un desarrollo adecuado. También surgieron dudas, incertidumbres y temores respecto a la calidad del entorno y la actuación de los educadores. Los relatos maternos expresaron que la educación y el estudio son vías para alcanzar el éxito en la vida, combinadas con la felicidad y la toma de buenas decisiones. Se destacó el enfoque en valores religiosos como una forma de creer que los niños estarán protegidos y seguirán el camino correcto hacia un futuro prometedor, basado en estas creencias.

Gran parte de las participantes informó un ingreso familiar inferior a tres salarios mínimos y, al abordar sus expectativas para el futuro de sus hijos, manifestaron preocupaciones sobre las circunstancias sociales en las que viven. Un estudio señala que las familias que dependen en gran medida de cuidadores no parentales, como abuelos u otras personas, expresan inquietudes sobre la calidad y el acceso a la educación infantil^(43)^. Las intervenciones preventivas en el ámbito de la atención primaria en salud infantil, con acceso universal y un alto nivel de participación, han demostrado un impacto en la reducción de disparidades y en la comprensión de los mecanismos mediante los cuales la pobreza puede generar barreras en la salud relacional entre madres, padres e hijos^(44)^. Es importante resaltar que las decisiones sobre el cuidado alternativo brindado por diferentes personas e instituciones pueden incidir positiva o negativamente en el desarrollo durante los primeros años de vida^(45)^.

En el presente estudio, el conjunto de resultados sobre las prácticas parentales maternas aportó elementos cualitativos para el desarrollo infantil, los cuales están interrelacionados con los dominios del *Nurturing Care*, en particular el cuidado responsivo, el aprendizaje oportuno y la seguridad y protección del niño, con un enfoque en su primer año de vida. Se destaca que estos dominios deben fortalecerse en los servicios de salud, con el objetivo de garantizar una atención de alta calidad, basada en valores centrados en las personas y que sea equitativa, resiliente y eficiente^(46)^. Por lo tanto, en los servicios de salud, las intervenciones parentales orientadas a la prevención de problemas y la promoción del desarrollo infantil son fundamentales en los primeros tres años de vida^(6,47-48)^. Los programas enfocados en mitigar las desventajas iniciales son esenciales para proporcionar estimulación responsiva y oportunidades de aprendizaje adecuadas^(49)^. Además, es necesario replantear las estrategias dentro del sistema de cuidados primarios para abordar las barreras estructurales, los desafíos organizativos y las limitaciones financieras^(50)^.

Este estudio presenta implicaciones para el cuidado de enfermería en la salud infantil en la APS, a partir de los resultados sobre las prácticas parentales en el hogar, con énfasis en el desarrollo de niños menores de un año, una etapa en la que ocurren muchos cambios y el cerebro en desarrollo es más sensible a las experiencias y al entorno.

El cuidado de enfermería en la salud infantil, en muchos momentos, debe identificar, abordar e intervenir en situaciones que incluyen: (i) cuidados parentales responsivos, para fomentar conversaciones, atención y afecto con el niño, analizar la rutina de cuidados, observar los hitos del desarrollo infantil, incentivar la proactividad paterna e identificar preocupaciones parentales sobre el desarrollo; (ii) aprendizaje desde una edad temprana, adecuado a cada etapa, para estimular a través de juegos, lecturas, canciones y paseos, y propiciar el contacto con otros niños; y (iii) seguridad y protección infantil, para equilibrar el consuelo del niño con el establecimiento de límites, prevenir accidentes domésticos y conocer los beneficios de una educación infantil de calidad. Por lo tanto, apoyar cuidados responsivos y un aprendizaje adecuado a la edad, así como intervenir en favor de la seguridad y protección del niño, configuran una actuación profesional prometedora y cualificada.

En la presente investigación, la atención a las situaciones de un mapa diario contribuye a identificar las vulnerabilidades y actuar para reducirlas, con base en los dominios del *Nurturing Care*. Así, las intervenciones oportunas, creativas y promotoras de la salud y del desarrollo infantil deben ser intrínsecas al cuidado de enfermería en salud infantil, en alianzas y corresponsabilidad parental para abordar fragilidades y ampliar un entorno adecuado de cuidado y desarrollo integral del niño. Los elementos cualitativos aquí analizados también refuerzan la necesidad de fortalecer la continuidad del cuidado, inherente a la atención en puericultura, en visitas domiciliarias, en el control prenatal y en la gestión del cuidado infantil, los cuales constituyen espacios clave en el cuidado de enfermería en salud infantil en la APS.

Las limitaciones del estudio se refieren a la realización de las entrevistas únicamente con las madres y enfoque en el primer año de vida de los niños, centradas en las especificidades de la realidad de un municipio de pequeño porte con unidades de ESF. Aunque no se abordaron los dominios del *Nurturing Care* relacionados con la buena salud y la nutrición adecuada, los resultados evidenciaron la relevancia de los cuidados responsivos, el aprendizaje oportuno, y la seguridad la protección en el primer año de vida, destacando tanto las potencialidades como las vulnerabilidades del desarrollo infantil en el contexto domiciliario. Otras investigaciones son necesarias para explorar las percepciones de otros cuidadores parentales y no parentales, así como para abordar diferentes grupos etarios y las adversidades en el desarrollo durante la primera infancia.

## Conclusión

Las prácticas parentales maternas fueron analizadas con énfasis en la percepción de la rutina diaria del niño orientada al desarrollo infantil, resaltando cualidades y fragilidades de su vida cotidiana. Además, se amplió la comprensión del entorno de cuidado e interacción, el cual proporciona oportunidades de aprendizaje y también presenta restricciones en el día a día, sugiriendo exponer a los niños a vulnerabilidades en momentos circunstanciales vitales para sus necesidades de desarrollo desde una edad temprana.

Los dominios del *Nurturing Care* relacionados con los cuidados responsivos, el aprendizaje oportuno y la seguridad y protección son fundamentales para el niño durante su primer año de vida, ya que reflejan experiencias de interacción, distintos momentos y acciones dentro del entorno de cuidado en el hogar. El enfoque en el primer año de vida permite comprender la dinámica del desarrollo infantil, indicando nuevas formas de percibir y guiar los cuidados parentales. Así, estos dominios permiten la identificación de circunstancias que evidencian tanto vulnerabilidades como potencialidades en el cuidado, dado su carácter contingente, ya que implica enfrentar eventualidades, incertidumbres y las diferencias inherentes a las experiencias vividas.

El enfoque del *Nurturing Care* ofrece dominios de análisis en el ámbito de la APS, reafirmando la importancia de considerar la responsividad parental en el cuidado infantil y de comprender su rutina diaria en aspectos esenciales para el desarrollo pleno y el cuidado integral, sugiriendo una estrategia promotora del cuidado de enfermería en salud infantil.
